# GENI as an AMPK Activator Binds α and γ Subunits and Improves the Memory Dysfunction of Alzheimer’s Disease Mouse Models via Autophagy and Neuroprotection

**DOI:** 10.3390/antiox14010057

**Published:** 2025-01-06

**Authors:** Ying Wang, Lanjie Li, Danni Chen, Jiaheng Shan, Meijuan Yi, Hiroyuki Osada, Minoru Yoshida, Lan Xiang, Jianhua Qi

**Affiliations:** 1 College of Pharmaceutical Sciences, Zhejiang University, Yu Hang Tang Road 866, Hangzhou 310058, China; 11819026@zju.edu.cn (Y.W.); lilanjie@zju.edu.cn (L.L.); 12019025@zju.edu.cn (D.C.); 22119073@zju.edu.cn (J.S.); 22119153@zju.edu.cn (M.Y.); 2 Chemical Biology Research Group, RIKEN Center for Sustainable Resource Science, wako, Saitama 351-0198, Japan; osadahiro@riken.jp; 3 Chemical Genomics Research Group, RIKEN Center for Sustainable Resource Science, Wako, Saitama 351-0198, Japan; yoshidam@riken.jp; 4 Jinhua Institute, Zhejiang University, Jinhua 321299, China

**Keywords:** iridoid glycoside, AMP-activated protein kinase, autophagy, neuroprotection, Alzheimer’s disease, target identification

## Abstract

Geniposidic 4-isoamyl ester (GENI) with anti-aging effects is a new iridoid glycoside derivative from *Gardenia jasminoides* Ellis found in our previous study. In this study, to indicate whether this compound has anti-Alzheimer’s disease (AD) effect, the galactose-induced AD mice and naturally aging mice with AD were used to do drug efficacy evaluation. Furthermore, the Western blot, small interfering RNA (siRNA), drug affinity responsive target stability (DARTS), cellular thermal shift assay (CESTA), liquid chromatography-tandem mass spectrometry (LC/MS-MS), adenosine 5′-monophosphate-activated protein kinase (AMPK) mutants and surface plasmon resonance (SPR) analysis were utilized to clarify the mechanism of action and identify target protein of this molecule. GENI exerts anti-AD efficacy in galactose-induced AD mice and naturally aging mice with AD through neuroprotection and modification of autophagy and neuron inflammation. Moreover, AMPK as the target protein of GENI to produce an anti-AD effect is identified and the ASP148, ASP157, and ASP166 of the AMPK α subunit and lysine (LYS)148, aspartic acid (ASP)156, LYS309, and ASP316 in the AMPK γ subunit as binding sites are confirmed. Meanwhile, the AMPK/unc-51-like autophagy-activating kinase 1 (ULK1)/microtubule-associated protein 1 light chain 3 beta (LC3B) and AMPK/mammalian target of rapamycin (mTOR) signaling pathways involved in anti-AD effects of GENI. The findings provide a new perspective on treating neurodegenerative diseases by activating AMPK for the energy metabolism disorder.

## 1. Introduction

AD is a progressive neurodegenerative disease that is the sixth leading cause of death and the most common cause of dementia worldwide [1]. Currently, at least 55 million patients in the world have AD, and this number is expected to reach around 139 million by 2050. The global cost of treatment and care has reached trillions of dollars every year, placing a heavy burden on patients’ families and society [2]. It is well known that the pathogenesis of AD includes cholinergic deficiency, amyloid beta (Aβ) accumulation, tau hyperphosphorylation, synaptic function disorders, oxidative stress, and neuroinflammation toxicity [3]. Some AD drugs, such as donepezil, huperzine A, rivastigmine, galantamine, aducanumab, and lecanemab which focus on the causes of these diseases have been developed and launched [4,5]. However, these drugs only can improve the clinical symptoms, but cannot cure this disease. Therefore, developing the drugs to treat AD is a challenging task for us.

In addition to the well-established treatment of AD by reducing Aβ levels, successful AD treatment strategies may require the concurrent application of neuroprotective agents [6]. Potential neuroprotective strategies include the elucidation of pathophysiological processes occurring in AD and the identification of molecular targets mediating these processes. Candidates of neuroprotective drugs have features of blocking the accumulation of oxidative stress products, such as reactive oxygen species (ROS), degradation of toxic proteins through autophagy, and inhibition of neuroinflammation [7]. Therefore, finding functional molecules that can simultaneously regulate multiple pathologies will be a viable strategy in the prevention and treatment of AD.

Some evidence has indicated that AMPK plays an important role in AD [8,9]. Activation of this protein can regulate tau protein phosphorylation through SIRT1 and GSK-β as well as reduce the expression level of APP beta secretase, thereby reducing Aβ production. Meanwhile, the amount of Aβ in the mouse neuron of AMPK knockout is significantly elevated [8,9]. On the other hand, abnormal energy metabolism is a common phenomenon in AD. The enzymes and proteins involved in cholesterol and low-density lipoprotein transport are closely related to the occurrence of Alzheimer’s disease [10]. At the same time, using positron emission tomography (PET) to study AD, it was found that the cerebral glucose metabolism rate of AD patients is significantly reduced, and the decline degree is positively correlated with the degree of cognitive impairment in patients [11]. However, AMPK activation can promote the expression of glucose transporter and transfer to the surface, and increase the expression of glucose transporter 3 (GLUT3) to promote glucose uptake by neurons [12]. Accordingly, AMPK agonists may be developed as new drugs for treating Alzheimer’s disease.

Iridoid glycosides, as a major class of natural products, have a wide range of pharmacological activities [13]. However, the identification of targets corresponding to the biological activity of iridoid glycosides is rarely reported. GENI is a compound that we previously discovered to exhibit significant anti-aging activity through the structure–activity relationship study of iridoid glycosides derived from *Gardenia jasminoides* Ellis (*G. jasminoides* Ellis) [14]. On the basis of the close relationship between aging and AD [15], we studied the anti-AD efficacy and mechanism of GENI with two kinds of AD animal models. Here, we report that this compound directly activated its target protein AMPK by binding at ASP148, ASP157 and ASP166 of the AMPK α subunit and LYS148, ASP156, LYS309 and ASP316 of the AMPK γ subunit to produce neuroprotective effects against AD, and the AMPK/ULK1/LC3B and AMPK/mTOR signaling pathways were involved in the anti-AD effect of GENI.

## 2. Materials and Methods

### 2.1. Cell Lines and Mice

PC12 cells were purchased from the National Collection of Authenticated Cell Cultures (Shanghai, China), and murine microglia (BV-2) were purchased from Cobioer Biosciences Co., Ltd., (Nanjing, China). Cells were routinely maintained at 37 °C in humidified air containing 5% CO_2_.

In animal experiments, sixty male ICR mice (7 weeks old, 30–35 g) were purchased from Zhejiang Academy of Medical Sciences (Hangzhou, China) and forty female C57BL/6j mice (19 months old, 29–32 g), and ten female C57BL/6j mice (8 weeks old, 29–32 g) were purchased from Biomice (Nantong, China), respectively. Five mice per cage were housed, allowed free access to water and food, and were maintained at constant temperature (23 ± 1 °C) and humidity (55% ± 5%) on a 12 h light/dark cycle (lights on 8:00 to 20:00). All experiments were performed in accordance with the Animal Experiment Committee of Zhejiang University (The animal ethical approval number: ZJU 20220084 and ZJU 20230049; the animal use permit number: SCXK (Zhejiang) 2019-0002 and SCXK (Jiangsu) 2021-0003).

### 2.2. Preparation of GENI

GENI was prepared according to the synthesis method we reported earlier [14]. The chemical structure of GENI was confirmed by comparing HR ESI-MS, ^1^H NMR, and ^13^C NMR data with our data.

### 2.3. Neuroprotection Assay of GENI on PC12 Cells

We seeded 5 × 10^4^ PC12 cells in each well of a 24-well plate and incubated the cells for 24 h at 37 °C in 5% CO_2_. The medium was changed to 1 mL of serum-free Dulbecco’s modified Eagle’s medium (DMEM; Thermo Scientific, Shanghai, China) containing the different test samples. To determine the optimal concentration of hydrogen peroxide (H_2_O_2_), we treated cells with 0.5% dimethyl sulfoxide (DMSO) for 24 h and then incubated the cells with different concentrations of H_2_O_2_ for 1 h. In this work, 0.8 mM H_2_O_2_ was selected as the best dose for the following experiment. To study the neuroprotective effects of GENI, we treated PC12 cells with resveratrol (RES 10 μM) or GENI (1, 3, or 10 μM) for 24 h and 0.8 mM H_2_O_2_ for 1 h. The medium was changed to 500 μL of serum-free DMEM containing 200 μg/mL 3-(4,5-dimethyltaizol-2-yl)-2,5-diphenyltetrazolium bromide (MTT) and incubated for an additional 2 h. The medium was completely removed, and 200 μL of DMSO was added to each well to dissolve the formed formazan crystals. The resulting formazan was detected at 570 nm using a microplate reader (Bio-Tec Instruments Inc., Winooski, VT, USA).

### 2.4. ROS, MDA, and, Total SOD Enzymatic Activity Assays in PC12 Cells

To determine ROS levels in PC12 cells, we seeded 5 × 10^4^ PC12 cells in each well of a 24-well plate. Cells were treated with RES (10 μM) or GENI (1, 3, and 10 μM) for 24 h, followed by 0.8 mM H_2_O_2_ for 1 h. DCFH-DA (2,7-dichlorodihydrofluorescein diacetate, final concentration, 10 μM) was then added to each well and incubated for 30 min. Cells were washed with PBS to remove extracellular DCFH-DA, and intercellular ROS was detected by using a SpectraMax M3 multimode microplate reader (Molecular Devices Corporation, San Jose, CA, USA) at an excitation wavelength of 488 nm and an emission wavelength of 525 nm. Meanwhile, DCF in PC12 cells was observed using a fluorescence microscope (HCS, Thermo Fisher, Science, Waltham, MA, USA). To detect malondialdehyde (MDA) content and total SOD enzymatic activities in PC12 cells, we seeded 2 × 10^6^ PC12 cells in 60 mm dishes containing 5 mL of DMEM and cultured the cells for 24 h. PC12 cells were treated with RES (10 μM) or GENI (3 or 10 μM) for 24 h and then treated with 0.8 mM H_2_O_2_ for 1 h to determine the MDA content and total superoxide dismutase (SOD) activities. Cells were lysed and centrifuged. Cell lysates were centrifuged, and supernatants were obtained to analyze MDA level and total SOD enzymatic activities. MDA quantification and total SOD activities were determined using MDA and SOD enzyme activity assay kits (Nanjing Jiancheng Bioengineering Institute, Nanjing, China) following the manufacturer’s instructions.

### 2.5. Real-Time Polymerase Chain Reaction (RT-PCR) Analysis

RT-PCR analysis was performed as described in the material and methods section in Supplementary Information.

### 2.6. Autophagy Flow Assay

The experiment was carried out according to the instructions of the CYTO-ID autophagy detection kit (Enzo Biochem, New York, NY, USA). In brief, we seeded 7.5 × 10^4^ PC12 cells in each well of a 24-well plate using the cell slide method. The cells were incubated for 24 h at 37 °C in a 5% CO_2_ incubator. The experiment was divided into control (0.5% DMSO), positive control rapamycin (Rap 500 nM + chloroquine (CQ)), GENI treated-group (10 μM GENI + 10 μM CQ), 3-MA inhibitor group (5 mM 3-MA + 10 μM CQ) and GENI + 3MA group (5 mM 3-MA + 10 μM GENI + 10 μM CQ). The optimum concentration of 3MA inhibitor was referenced in the other report [16]. At first, 0.5 mL of serum-free DMEM (plus 1% dual anti-penicillin–streptomycin) containing 3-MA (5 mM) was used to replace the medium in two groups as well as 0.5 mL of serum-free DMEM was added in the other groups, respectively. After treating for 6 h, 0.5 mL of DMEM containing DMSO (0.5%), Rap (500 nM) + CQ (10 μM), GENI (10 μM) + CQ (10 μM), CQ (10 μM), and GENI (10 μM) + CQ (10 μM) was added into the corresponding group, respectively. After 18 h, we removed the medium and washed cells twice with 1× assay buffer containing 5% FBS. We then added CYTO-ID Green detection reagent and Hocchst 33342 nuclear stain to the cells, which were incubated at 37 °C for 45 min. After three washes with 1× assay buffer, we fixed cells with 4% paraformaldehyde for 20 min. After three washes with 1× assay buffer, we observed the cells with an upright two-photon confocal microscope (Olympus FV1000BX-51, Tokyo, Japan).

### 2.7. BV-2 Microglial Activity Assay

BV-2 cells (2.5 × 10^4^) were seeded in 24-well plates. After treatment with 500 μL of RPMI 1640 medium (plus 1% dual anti-penicillin–streptomycin) containing 1, 3, and 10 μM GENI for 2 h, 500 μL of medium containing lipopolysaccharide (LPS 1 μg/mL) was added for 24 h. We then removed the medium, washed the cells three times with PBS, and fixed the cells with 4% paraformaldehyde (500 μL/well) for 20 min at room temperature. We washed the cells three times with PBS and added Quick Block^TM^ immunostaining blocking solution (500 μL/well) to block for 60 min. After removing the blocking solution, we added 200 μL of the primary antibody (Anti-Iba1 rabbit monoclonal antibody, 1:1000, abcam178847) to each well and incubated the samples overnight at 4 °C. The next day, the supernatant was discarded, washed three times with PBS, and incubated with the secondary antibody Cart anti-rabbit IgG (1:1000) at room temperature for 1 h. Nuclei were stained with DAPI (250 μL/well) for 5 min, and the fluorescence was observed under a fluorescent inverted microscope (Olympus IX53, Tokyo, Japan).

### 2.8. Enzyme-Linked Immunosorbent Assay

ELISA analysis for NO and interleukin-6 (IL-6) was performed according to the manufacturer described in the material and methods section in Supplementary Information.

### 2.9. Animal Experiment

In the animal experiment of D-galactose (D-gal) induced AD mice, D-gal was used to induce AD model of mice [17] and donepezil (DON) as the first-line medicine for treating AD was selected as positive control [18]. The mice were randomly divided into the following groups: normal control group, 450 mg/kg D-gal group, D-gal + 3 mg/kg DON group, D-gal + 12.5 mg/kg GENI group, D-gal + 25 mg/kg GENI group, and D-gal + 50 mg/kg GENI group. Each group comprised 10 mice. DON and GENI were dissolved in ultrapure water and administered by gavage, whereas D-gal was dissolved in normal saline and administered by intraperitoneal injection. Dosing lasted for 10 weeks. Behavioral experiments including Y-maze, novel object recognition (NOR) test, and Morris water maze (MWM) test started after the 8th week of administration and lasted for 2 weeks. The mice were sacrificed after 10 weeks of administration. Blood was collected for biochemical index analysis, and the hippocampus, cerebral cortex, and liver were collected for Western blot and immunohistochemical staining experiments.

In experiments with a naturally aged mouse model of AD, the mice were randomly divided into the following groups: normal young control group, normal aged control group, aged + 25 mg/kg GENI, aged + 2 mg/kg dorsomorphin (Dor) and aged + 2 mg/kg Dor + 25 mg/kg GENI. Each group had 10 mice. GENI was dissolved in ultrapure water and administered by gavage, whereas Dor was dissolved in normal saline. The pH was adjusted to pH 5 with 0.1 M hydrochloric acid to the clarified solution and administered by intraperitoneal injection. The dosing experiment also lasted 10 weeks. Animal behavioral experiments and sampling at the end of the animal experiment were performed as described above.

### 2.10. Animal Behavior Test

The Y-maze is mainly used to study the spatial working memory ability of rodents. The Y-maze consisted of three equal-length arms (50 cm × 18 cm × 35 cm), and the angle between each two arms was 120 degrees. The mice were placed at the end of any arm of the Y-maze and allowed to explore freely for 8 min. The behavioral changes including the total number of entries and alternation (successively entering all three arms of the Y-maze once in turn) were recorded. Percentage altercation = alternation/(entries-2) × 100%. The Y-maze could effectively reflect the ability of animals to recognize and remember novel environments.

NOR was used to detect memory in rodents. We prepared a 40 × 40 × 40 cm square box, camera equipment, and behavioral analysis software. We chose three objects A, B, and C, where objects A and B were exactly the same, and object C was very different from objects A and B. After the start of the experiment, the mice were allowed to acclimate freely in the experimental apparatus for 10 min. Objects A and B were placed into the box, and the mice were allowed to explore for 5 min. We then recorded the exploration time and times. After 1 h, A and C or B and C were placed into the box. After letting the mice explore for 5 min, we recorded the exploration time of the original object and the new object. Discrimination index = exploration time of new objects/total exploration time × 100%.

The MWM installation consisted of a circular pool (120 cm in diameter and 50 cm in height), camera equipment, and a computer analysis system. In the first stage, the platform position was fixed, and mice were placed into the pool from any three of the four origins on the pool facing away from the pool wall. The time when it took for the mouse to find the platform within 120 s, called escape latency, was recorded. The training period lasted 4 days. The escape latency of the mice was recorded daily, and the average value was used as an index to evaluate the ability of learning and memory. On the 5th day, the platform was evacuated by the experimenter. The mice were placed against the platform into the water from any water entry point, and they were allowed to swim freely for 60 s. We then recorded the number of times that they crossed the platform.

The rationale for the three-box social test is based on the fact that mice are naturally gregarious and tend to explore new objects. The experimental device consisted of three rectangular boxes, each with a size of 19×45 cm, with a channel in the middle to allow the three boxes to connect with one another. Two metal cages of the same size were placed in the centre of the left and right boxes. Firstly, the mice were placed in the box for 10 min. Secondly, stranger 1 mouse was randomly placed in the metal cage in the left or right box, and the metal cage in the other box was empty. The experimental mice were placed in the box for 10 min. The number and duration of direct contact between the experimental mice and stranger 1 were recorded. Thirdly, strange 2 mice were placed in the empty metal cage of the second stage experiment and then recorded for 10 min. We observed the time and number of contacts between stranger 1 and stranger 2 and calculated the social preference and sociability index.

### 2.11. Immunohistochemistry

Firstly, the three mice of each group were subjected to heart perfusion at the end of the animal experiment, and the whole brain was removed and placed in 15 mL of 4% paraformaldehyde and stored at 4 °C for 24 h. Secondly, we changed 4% paraformaldehyde into 15% sucrose solution and stored the solution at 4 °C for 24 h. We then changed the solution to 30% sucrose solution to dehydrate for 2 days. Thirdly, the mouse brain tissue was embedded in a cryo-embedding medium, and about 80 slices with a thickness of 20 μm were cut in the hippocampus using a cryostat (Thermal Fisher, Shanghai, China). The sections of mice in each group were immunohistochemically stained with primary antibodies, such as neuronal nuclear anti gen (NeuN), ionized calcium-binding adapter molecule 1 (Iba-1), inducible nitric oxide synthase (iNOS, pro-inflammatory factors), synaptophysin (Syp), and glial fibrillary acidic protein (GFAP), and secondary antibody. The product number and usage ratios of primary and secondary antibodies are detailed in Supplementary Table S4. Finally, the sections were observed under an upright two-photon confocal microscope (Olympus BX61, Tokyo, Japan) and recorded by a camera. The pictures were digitized with ImageJ software V1.8.0.112 (National Institutes of Health, Bethesda, MD, USA).

### 2.12. Western Blot Analysis

Western blot analysis for p-AMPK α, AMPK α, p-mTOR, mTOR, p-ULK1, ULK1, and LC3-I/II was conducted according to the information described in the materials and methods section of Supplementary Information.

### 2.13. Cellular Thermal Shift Assay

In a 60 mm dish containing 5 mL of DMEM, 2 × 10^6^ cells were added and incubated for 24 h. In each plate, 10 μM GENI was added and incubated for 2 h. Cells were harvested and heated at a temperature ranging from 60 °C to 80 °C for PC12 cell samples. We obtained a certain amount of cerebral cortex and hippocampus samples, which were added with PBS and ground. After centrifugation, the supernatant was used as a sample, and we measured the protein concentration with a BCA kit (CoWin Biotech Co., Ltd., Taizhou, Jiangsu, China). The protein of the cerebral cortex or hippocampus was incubated with 10 μM GENI at 37 °C for 2 h and heated from 65 °C to 100 °C for the cerebral cortex sample but 43 °C to 67 °C and 65 °C to 100 °C for the hippocampus sample. Equal volumes of supernatant heated at each temperature point for the control and treatment groups were used for Western blot analysis with the antibody for specific proteins.

### 2.14. RNA Interference

PC12 cells were transfected with different concentrations of FAM-labeled siRNA to evaluate transfection efficiency. A concentration of 80 nM was determined as the final concentration for the experiment to obtain a transfection efficiency of 90%. The following primer sequences were used to generate AMPK α knockdown and negative control siRNA (Sangon Biotech Co. Ltd., Shanghai, China): For AMPK α, sense: 5′-GCA CCC UCA UAU AAU CAA ATT-3′, anti-sense: 5′-UUU GAU UAU AUG AGG GUG CTT-3′; for negative control, sense: 5′-UUC UCC GAA CGU GUC ACG UTT-3′, antisense: 5′-ACG UGA CAC GUU CGG AGA ATT-3′. Transfection of PC12 cells with siRNA was performed according to the manufacturer’s instructions. In brief, we seeded 8 × 10^4^ cells in each well of a 24-well plate. Cells were allowed to reach 70–90% confluence in a growth medium without antibiotics the day before transfection. The siRNA against AMPK α or negative control siRNA was then used at a concentration of 80 nM, and Lipofectamine 2000 (Invitrogen, Shanghai, China) was used as the transfection agent. At 6 h after transfection, the previous medium in the plate was replaced with fresh medium containing 10 μM GENI, and the plate was incubated for an additional 2 h using Western blot analysis to detect changes in AMPK α protein.

### 2.15. Inhibitor Experiment

Firstly, 2 × 10^6^ cells were added to a 60 mm Petri dish containing 5 mL of DMEM and incubated for 24 h. Secondly, the C group, GENI group, Dor group, and GENI+ Dor group were established. The dishes containing the Dor group were pre-treated with 5 μM Dor for 2 h, and the remaining groups were balanced with DMSO. The group containing 10 μM GENI was added, and the remaining groups were balanced with DMSO. After incubation for 2 h, protein extraction was performed for the Western blot experiment.

### 2.16. Drug Affinity Responsive Target Stability

Cells were lysed by RIPA lysis buffer, and the protein concentration was measured. The lysate was divided into six groups. Before incubation with protease (protein: pronase = 50:1 *w*/*w* for PI3K and AMPK detection, 5:1 for MAPK detection, 100:1 for p53 detection) for 25 min in 25 °C, 0.1–100 μM GENI was added to lysates and incubated for 2 h at room temperature. The samples were mixed with loading buffer and then separated on 8% SDS-PAGE for immunoblotting analysis.

### 2.17. AMPK Purification

Mammalian AMPK subunits (His-α1, β1, and γ1) were cloned into a pET-3d vector, and protein expression and purification were similar to the literature [19].

### 2.18. Surface Plasmon Resonance Analysis

SPR analysis was performed with a Biacore T200 instrument (GE Healthcare). Firstly, the purred His-tagged AMPK containing α1β1γ1 protein was captured onto an activated carboxymethylated 5 (CM5) chip using amine coupling to levels ranging from 4000 RU to 6000 RU. Secondly, GENI was dissolved in PBS-P buffer solution (0.05% (*v*/*v*) Tween 20 and PBS), and the concentration of GENI was 64 μM. Thirdly, the solution of GENI was diluted to 32 μM, 16 μM, 8 μM, 4 μM, 2 μM, 1 μM and 0.5 μM with PBS-P buffer solution. The sample was filtered by pumping through a 0.2 mm membrane. A gradient concentration of GENI was injected at a flow rate of 30 μL/min in a running buffer (0.05% (*v*/*v*) Tween 20 and PBS). The data were analyzed with Biacore evaluation software (T200 version 2.0) and fitted to the 1:1 Langmuir binding model, and the kinetic parameters were derived.

### 2.19. Determination of GENI-Binding Sites on AMPK

Recombinant AMPK protein was incubated with GENI (10 μM) or DMSO with rotation at 4 °C overnight. Reactions were ceased by adding a loading buffer, and proteins were separated by 8% SDS-PAGE. After visualization by Coomassie blue staining, the bands corresponding to AMPK, and subunits were cut and digested with trypsin.

The binding sites were identified by LC-MS/MS using Mass Spectrometer LTQ Orbitrap Elite. The peptide mixture was injected onto the capture column using Thermo Scientific Easy nanoLC 1000 at a flow rate of 10 μL/min and held for 2 min. The trap is balanced to a maximum pressure of 500 bar at 12 μL, followed by column balancing at a maximum pressure of 500 bar at 3 μL, and then gradient elution of the column is initiated. 5-step linear gradient elution (A: dd H_2_O + 0.1% formic acid, B: ACN + 0.1% formic acid) was performed: 0–10 min, 3–8% B; 10–120 min, 8–20% B; 120–137 min, 20–30% B; 137–143 min, 30–90% B; 143–150 min, 90% B). Column flow was maintained at 250 nL/min. The chromatographic system consisted of A trap column (75 μm × 2 cm, nanoviper, C18, 3 μM, 100 A) and an analysis column (50 μm × 15 cm, nanoviper, C18, 2 μM, 100 A). Nanspray Flex ionization source and FTMS (Fourier transform ion cyclotron resonance mass) equipped with Thermo LTQ-Orbitrap Velos Pro were adopted. Data are collected in conjunction with the ion Trap analyzer equipped with the Thermo LTQ-Orbitrap Elite. The positive ion beam technique was used to analyze trace elements in the samples and was compared with the traditional analysis method. FTMS parameters are as follows: full scan mass spectrometry, data are collected at 60 K, the positive electrode is polarity, the profile is a data type, and then CID (1.0 *m*/*z* isolation width, 35% collision energy, 0.25 activation Q, 10 ms activation time) is used to separate the first 20 ions for MS/MS detection. For each sample, the standard curve or reference peak should be compared to analyze its characteristic information and determine the best measurement conditions. The scanning range is set to 300 *m*/*z* first mass and 2000 *m*/*z* final mass. The single-factor analysis of variance (ANOVA) was used to investigate the variation in the concentration of each component under different conditions. The parameters of the Ion Trap analyzer are within the normal mass range, the scanning rate is fast, and the type of centroid data are available.

### 2.20. Statistical Analysis

Data were evaluated by one-way ANOVA, followed by Tukey’s post hoc test by using GraphPad Prism software 8.0 (GraphPad Software, San Diego, California, USA). *p* value < 0.05 was considered statistically significant. Each experiment was repeated thrice, and data were expressed as mean ± SEM.

## 3. Results

### 3.1. GENI Exerted Neuroprotection Effects Through Anti-Oxidative Stress, Autophagy-Induction and Anti-Neuroinflammation Signaling Pathways

**Figure 1:**
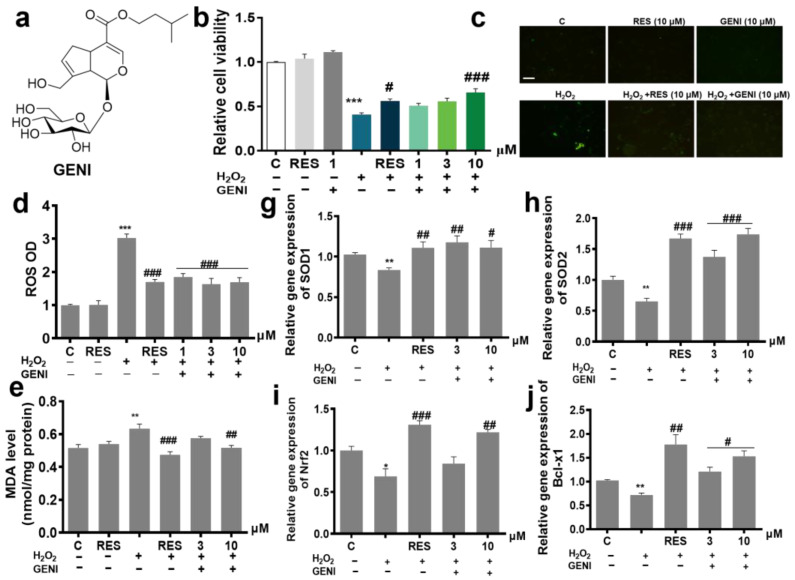

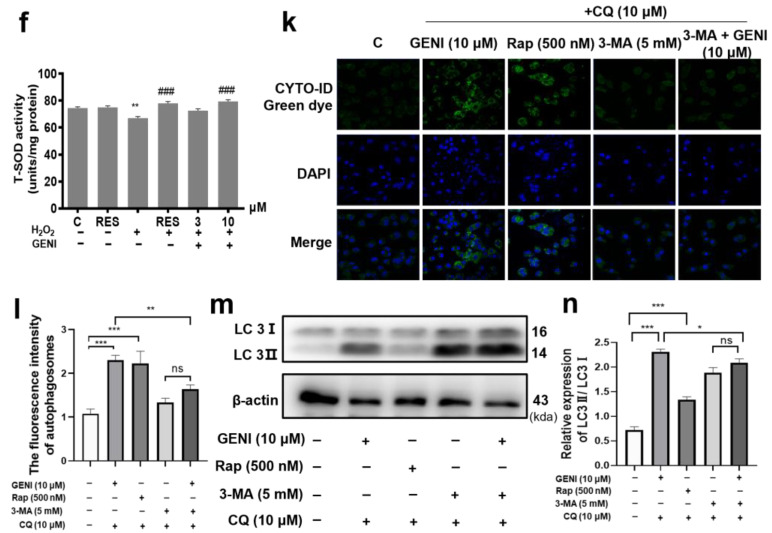
Neuroprotection activity of GENI through anti-oxidative stress and autophagy in vitro. (**a**) The chemical structure of GENI. (**b**) GENI exhibited neuroprotection on the H_2_O_2_-induced oxidative damage in PC12 cells. Resveratrol (RES) was used as a positive control (10 μM). (**c**) Microphotograph of PC12 cells stained with DCFH-DA to detect ROS under fluorescence microscopy. Scale bar, 100 μm. (**d**) Effect of GENI on the ROS of PC12 cells under oxidative stress conditions induced with or without H_2_O_2_. The levels of MDA content (**e**) and total SOD activity (**f**) of PC12 cells after treatment with GENI. PC12 cells were pre-treated with RES (10 μM) and different concentrations of GENI (3 and 10 μM) for 24 h and then treated with H_2_O_2_ (0.8 mM) for 1 h or with RES (10 μM) and GENI (3 and 10 μM) alone for 24 h. (**g**–**j**) SOD1, SOD2, Nrf2 and Bcl-x1 genes expression in PC12 cells under H_2_O_2_ induction. RT-PCR assays were performed after adding samples for 24 h. Data were presented as the mean ± SEM. * *p* < 0.05, ** *p* < 0.01 and *** *p* < 0.001 represented a significant difference from the blank control group; # *p* < 0.05, ## *p* < 0.01 and ### *p* < 0.001 represented a significant difference from the H_2_O_2_ treated group. (**k**,**l**) Effect of GENI on autophagy flux and effect of autophagy inhibitor, 3-MA on GENI-induced autophagy. Rap was used as a positive control (0.5 μM). CQ (10 μM) was used as a blocker of autophagic flux. 3-MA inhibitor (5 mM) was used to inhibit the conversion of LC3 I to LC3 II. (**m**,**n**) Western blot of LC 3 II/LC 3 I for the effect of GENI on autophagy flux and analysis of the protein level of LC 3 II/LC 3 I. Scale bar 100 μm. n = 3 independent experiments. Data were presented as the mean ± SEM. *** *p* < 0.001 represented a significant difference from the blank control group; * *p* < 0.05 and ** *p* < 0.01 represented a significant difference from the GENI-treated group; ns represented no significant difference from the 3-MA treated group.

Brain tissue has high oxidative metabolic activity, and strong production of reactive oxygen metabolites, so the brain is vulnerable to excessive oxidative damage [20]. Control of oxidative stress is a strategy for the treatment of neurodegenerative diseases. We found that GENI (Figure 1a) could significantly improve the survival rate of PC12 cells induced by H_2_O_2_ and play a neuroprotective role (Figure 1b); therefore, its mechanism of action was explored. ROS and MDA are two biomarkers of oxidative stress in cells. Therefore, we detected the changes in ROS and MDA in PC12 cells after treatment of GENI. GENI could significantly reduce the H_2_O_2_-induced increase in ROS and MDA in PC12 cells (Figure 1c,d, *p* < 0.001, *p* < 0.001, *p* < 0.001; Figure 1e, *p* < 0.01, *p* < 0.05), respectively. SOD is an important endogenous free radical scavenger in mammalian cells. The total SOD activity and the genes expression of SOD1 and SOD2 were increased in GENI-treated cells compared with that of the H_2_O_2_-treated group (Figure 1f, *p* < 0.001; Figure 1g, *p* < 0.01, *p* < 0.05; Figure 1h, *p* < 0.001, *p* < 0.001), respectively. In addition, Nrf2 plays an important role in protecting against oxidative stress and apoptotic damage. Bcl-x1, an anti-apoptotic protein, plays a considerable role in the resistance to apoptosis and neuroprotection. The abundance of Bcl-x1 and Nrf2 mRNA also significantly increased after GENI treatment (Figure 1i, *p* < 0.01; Figure 1j, *p* < 0.05, *p* < 0.05). In summary, GENI exerted anti-oxidative stress effects in PC12 cells by reducing the levels of ROS and MDA by upregulating the gene expression levels of SOD1, SOD2, Nrf2, and Bcl-x1 and increasing the activities of T-SOD.

Autophagy is an important and conserved lysosomal degradation pathway that controls cytoplasmic quality by eliminating intracellular aggregates and damaged organelles to maintain neuronal homeostasis. Subsequently, we explored whether the neuroprotective effect of GENI in PC12 cells is due to autophagy induction. Compared with the control group, the fluorescence intensity of autophagosomes in the GENI group pretreated with CQ significantly increased, and the effect of GENI at 10 μM was comparable with that of positive control Rap at 500 nM, which showed that GENI promoted autophagy. Meanwhile, the autophagy induced by GENI was inhibited by the autophagy inhibitor, 3-MA (Figure 1k,l, *p* < 0.001, *p* < 0.001). Furthermore, the changes in the LC3 I/II at the protein level after treatment of GENI and 3-MA were also detected (Figure 1m,n, Supplementary Figure S1). As we expected, the LC3 II at the protein level in the GENI group was significantly increased compared with the control group. After treatment of 3-MA, the conversion of LC3 I to LC3 II induced by GENI was diminished by this inhibitor.

In addition, inhibition of neuroinflammation, including the overactivation of glial cells and the expression of inflammation-related factors, may be one of the most effective strategies in the treatment of neurodegenerative diseases. The results showed that GENI exerted a potential anti-inflammatory effect by inhibiting LPS-induced activation in BV-2 microglia (Extended Data Figure S1a,b). Meanwhile, the contents of inflammatory factors in the supernatant of BV-2 cell culture after LPS stimulation and GENI treatment were also analyzed. GENI significantly reduced the secretion of LPS-stimulated pro-inflammatory IL-6 and the production of NO in BV-2 cells, and it displayed anti-neuroinflammatory activity (Extended Data Figure S1c,d). Thus, GENI exerted neuroprotection effects through anti-oxidative stress, autophagy induction, and anti-neuroinflammation.

### 3.2. GENI Ameliorated Memory Loss and Cognitive Impairment in D-Gal-Induced AD Mice

**Figure 2:**
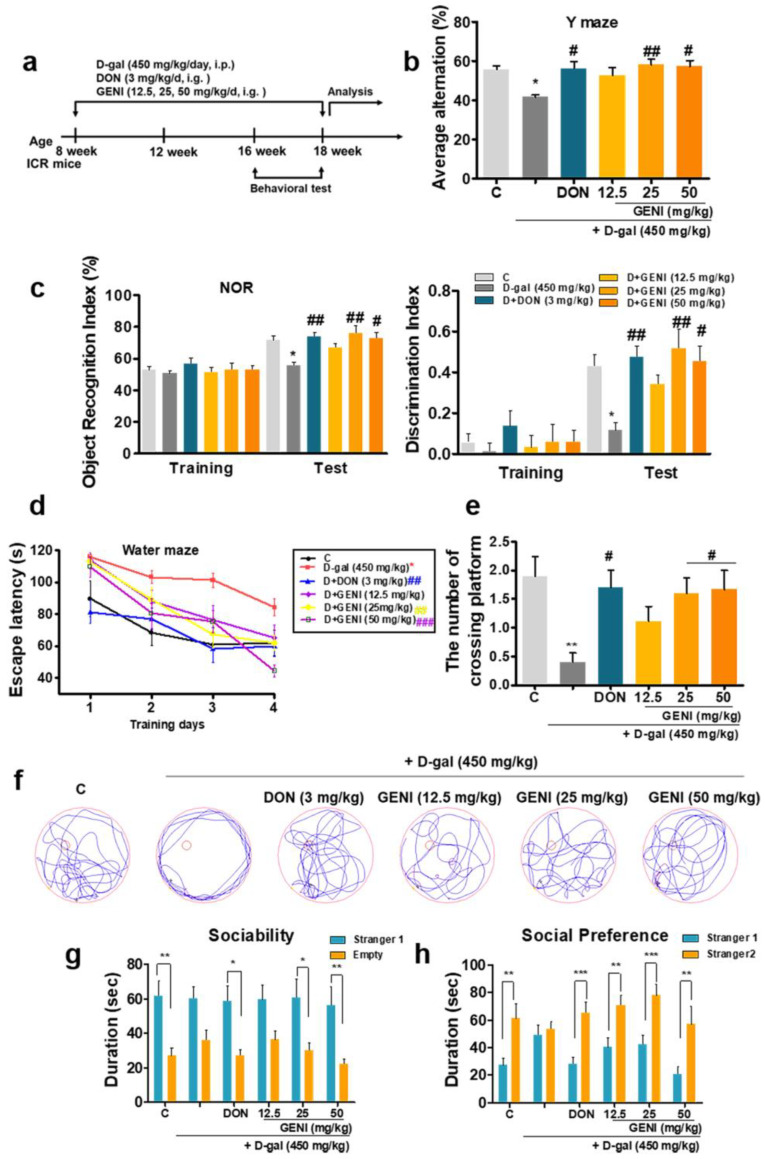
GENI ameliorated cognitive dysfunction in D-gal-induced AD mice. (**a**) The scheme of animal experiments. (**b**) Average alternation of the mice in the Y-maze experiment. (**c**) Changes in the recognition index and discrimination index of mice in the NOR test. (**d**) Changes in escape latency of mice in each group on training phase days 1–4 in water maze test. (**e**) Number of crossing platforms of mice in each group on test phase day 5 in the water maze. (**f**) Route diagram of crossing platform of mice on test day 5 in the water maze. (**g**,**h**) Changes in the sociability and social preferences of mice in the three-box social experiment. n = 10 mice. Data were presented as mean ± SEM. * *p* < 0.05, ** *p* < 0.01, and *** *p* < 0.001 represented a significant difference from the blank control group; # *p* < 0.05 and ## *p* < 0.01 represented a significant difference from the D-gal treated group.

To evaluate whether GENI has anti-AD effects, AD mice were constructed by continuous intraperitoneal injection of D-gal at a dose of 450 mg/kg/day for 2 months as well as GENI was orally administered at doses of 12.5, 25, and 50 mg/kg/day (i.g.), respectively. DON at a dose of 3 mg/kg, which is the first-line clinical drug for AD treatment was used as a positive control (Figure 2a). After administering GENI for 2 months, we performed animal behavioral experiments to evaluate the changes in memory of mice. The results of Y-maze, NOR and MWM showed that the mice of the D-gal model group demonstrated cognitive decline and memory impairment, which indicated the successful induction of the AD model (Figure 2b–h). GENI in the 25 and 50 mg/kg administration groups could significantly increase the rate of alternating arms in the Y maze test (Figure 2b, *p* < 0.01, *p* < 0.05). The cognitive index and object recognition index in the NOR experiment significantly increased, which revealed that GENI significantly improved the learning and spatial memory of AD mice (Figure 2c, *p* < 0.01, *p* < 0.05). In the Morris water maze test, the escape latency of the GENI administration group was significantly reduced compared with that of the D-gal model group, and the number of crossing platforms significantly increased (Figure 2d, *p* < 0.01, *p* < 0.001; Figure 2e,f *p* < 0.05, *p* < 0.05). During sociability analysis, the mice in the GENI-administered groups showed significant social skills and social preferences (Figure 2g, *p* < 0.05, *p* < 0.01; Figure 2h, *p* < 0.01, *p* < 0.001, *p* < 0.01). The above results indicated that GENI could rescue the cognitive deficit and memory impairment of D-gal-induced AD mice, thereby improving learning and working memory, as well as social behavior.

### 3.3. GENI Exerted Neuroprotection by Promoting Neuronal Repairment and Anti-Neuroinflammation in D-Gal-Induced AD Mice

**Figure 3:**
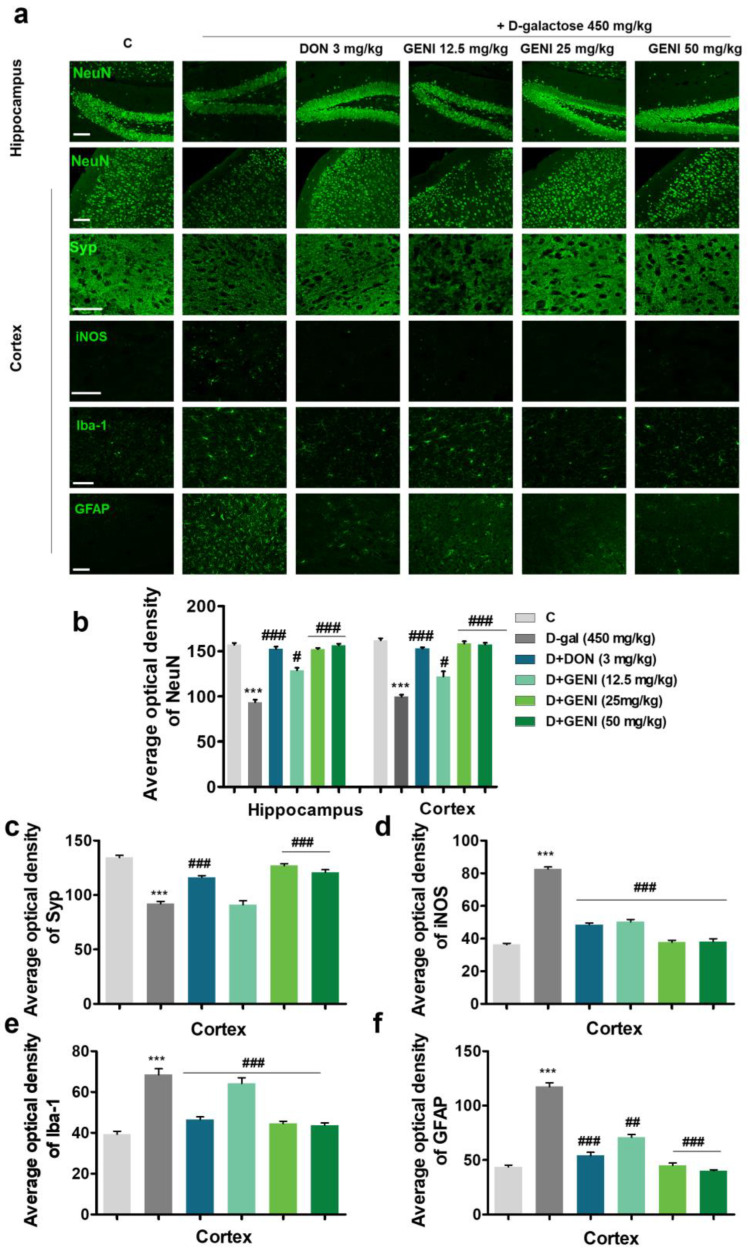
Neuroprotection effects of GENI on D-gal-induced AD mice. (**a**) Effect of GENI on NeuN in the hippocampus and cerebral cortex, and Syp, iNOS, Iba-1, and GFAP in the cerebral cortex. Scale bar, 100 μm. (**b**–**f**) Statistical results of the (**a**). n = 7 pictures from three mice. Data were presented as mean ± SEM. *** *p* < 0.001 represented a significant difference from the blank control group; # *p* < 0.05, ## *p* < 0.01 and ### *p* < 0.001 represented a significant difference from the D-gal treated group.

On the basis of the neuroprotective activity of GENI at the cellular level, the neuroprotective pharmacodynamics of GENI was evaluated at the animal level. Neurons are the basic units that constitute the structure and function of the nervous system, and synaptic transmission is essential for nervous system function. We performed immunohistochemical staining to detect neuronal and synaptophysin changes in the cerebral cortex and hippocampus (Figure 3a–c). Compared with the normal control group, the number of cortical and hippocampal mature neurons (NeuN) in the D-gal group was significantly reduced (Figure 3a,b, *p* < 0.001, *p* < 0.001), and the Syp intensity in the cerebral cortex was decreased (Figure 3a,c, *p* < 0.001). The GENI-treated groups and Don-treated group exhibited significantly increased mature neurons in the cerebral cortex and hippocampus compared with the D-gal group (Figure 3a,b, *p* < 0.05, *p* < 0.001, *p* < 0.001), and Syp intensity also significantly increased (Figure 3a,c, *p* < 0.001, *p* < 0.001, *p* < 0.001). In addition, the pathogenesis of AD was closely related to neuroinflammation [21]. The production of inflammatory cytokines and the expression of iNOS in the brains of patients with AD increased. The formation and appearance of Aβ plaques in the brain were associated with overactivation of microglia and astrocytes. Subsequently, we investigated the expression of iNOS, the changes in biomarkers of microglia, and astrocytes, Iba-1 and GFAP after GENI treatment, respectively. The expression of iNOS and activation of microglia and astrocytes in the cerebral cortex of the D-gal group significantly increased, respectively (Figure 3a,d, *p* < 0.001; Figure 3a,e, *p* < 0.001; Figure 3a,f, *p* < 0.001). However, these parameters in AD mice decreased after GENI and DON treatment (Figure 3a,d, *p* < 0.001, *p* < 0.001, *p* < 0.001; Figure 3a,e, *p* < 0.001, *p* < 0.001, *p* < 0.001, *p* < 0.001; Figure 3a,f, *p* < 0.01, *p* < 0.001, *p* < 0.001, *p* < 0.001). These results revealed that GENI like DON exerted neuroprotective effects by reducing the overactivation of microglia and astrocytes, and decreasing the release of pro-inflammatory factors, thereby improving memory dysfunction in D-gal-induced AD mice.

### 3.4. Screening Target Protein Candidates, Target Protein Identification and Exploration of the Signaling Pathways of GENI

**Figure 4:**
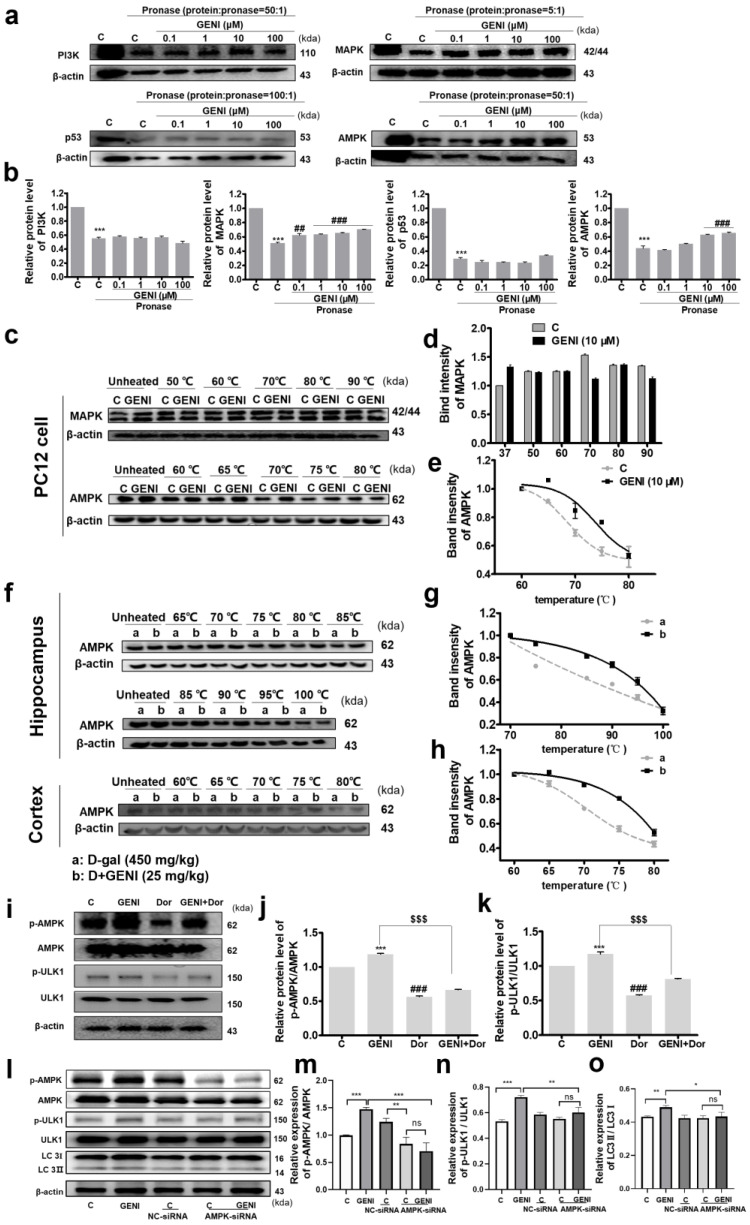
Screening candidates of target proteins of GENI. (**a**,**b**) Target protein candidates of GENI in DARTS analysis. The ratio of protease to protein was determined by pre-experiments as 50:1, 5:1, 100:1, and 50:1 (*w*/*w*), respectively. Data were presented as mean ± SEM. *** *p* < 0.001 represented a significant difference from the control group without pronase, ## *p* < 0.01, and ### *p*< 0.001 represented a significant difference from the control group treated with pronase. (**c**–**e**) The results of CETSA analysis for GENI and MAPK or AMPK proteins. (**f**–**h**) CETSA analysis for AMPK protein of hippocampus and cerebral cortex tissue and corresponding fitting curves. The samples of the control group and GENI treatment group were from D-gal model mice and D-gal model mice treated with GENI at 25 mg/kg. (**i**–**k**) Effect of Dor, an inhibitor of AMPK, on the effect of GENI in PC12 cells. Pre-treatment with the AMPK inhibitor Dor (500 nM) on PC12 cells for 30 min, treatment with GENI for 2 h, and analysis of the protein level of *p*-ULK1. n = 3 independent experiments. Data were presented as mean ± SEM. ** *p* < 0.01 and *** *p* < 0.001 and ### *p* < 0.001 represented a significant difference from the blank control group; $$$ *p* < 0.001 represented a significant difference from the GENI-treated group. (**l**–**o**) Effect of siRNA of AMPK on the effect of GENI in PC12 cells. Western blot analysis of p-AMPK, AMPK, p-ULK1, ULK1, LC3I/II after transfection with negative siRNA (NC siRNA) or AMPK siRNA, and treatment with GENI. Cells were transfected with lipofectamine 2000 and 80 nM siRNA for 6 h and then treated with GENI. n = 3 independent experiments. Data were presented as mean ± SEM. * *p* < 0.05, ** *p* < 0.01 and *** *p* < 0.001 represented a significant difference from the GENI-treated group, ns represented no significant difference from the GENI-treated group with AMPK-siRNA.

Identifying drug–target interactions is important for developing new drugs and understanding their side effects. To understand the mechanism of GENI for promoting autophagy, we screened the potential targets of GENI from several well-known autophagy targets at the cellular level. Several key target proteins of autophagy induction including phosphatidylinositol-3 kinase (PI3K), tumor suppressor protein (p53), mitogen-activated protein kinase (MAPK), and AMPK were examined by DARTS technique, firstly [22]. GENI dramatically enhanced the stability of MAPK and AMPK but had no obvious effect on other autophagy target proteins, PI3K and p53 (Figure 4a,b, Supplementary Figure S2). Subsequently, the CETSA method was used for further confirmation of MAPK and AMPK, and the experimental results showed that GENI could sensibly increase the thermal stability of AMPK, but the effect on MAPK was not obvious (Figure 4c–e, Supplementary Figure S3). These results clarified that AMPK may be the target protein of GENI. Subsequently, we confirmed whether AMPK is a target protein of GENI in the cerebral cortex and hippocampus of mice brains via CETSA analysis. GENI enhanced the stability of AMPK proteins in both the cerebral cortex and hippocampus in the brain (Figure 4f–h, Supplementary Figure S4). To confirm whether AMPK is the drug target of GENI for anti-AD effect, the AMPK inhibitor, Dor, and siRNA of AMPK were employed to competitively inhibit or interfere with AMPK activity at the cellular level. The results indicated that the phosphorylation of AMPK, ULK1, and LC3 II after GENI treatment was significantly decreased after treatment with Dor (Figure 4i–k, Supplementary Figure S5) and siRNA of AMPK (Figure 4l–n, Supplementary Figure S6). These results further confirmed that AMPK was a potential target of GENI.

**Figure 5:**
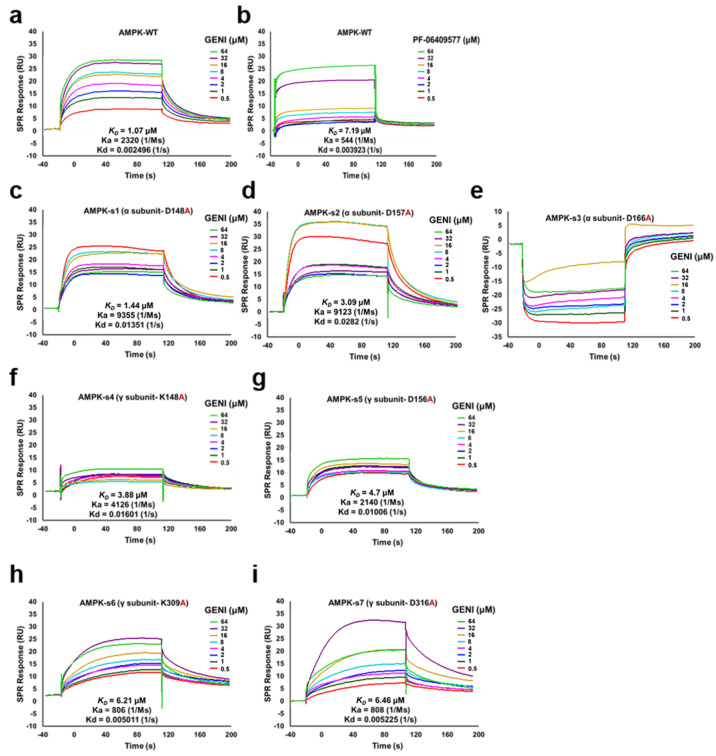
Interaction of AMPK and GENI, and binding sites confirmation of GENI with wild-type and mutants of AMPK by SPR analysis. (**a**,**b**) SPR analysis of GENI and PF-06409577 with purified AMPK protein of wild-type. (**c**–**e**) ASP148, 157, and 166 of AMPK α subunit were mutated into ALA148, 157, and 166, and the binding of AMPK mutants and GENI was measured by SPR. (**f**–**i**) LYS148, ASP156, LYS309, and ASP316 of AMPK γ subunit were mutated into ALA148, ALA156, ALA309 and ALA316, and the binding of AMPK mutants and GENI was measured by SPR. The contact and dissociation time were 120 s and 240 s, respectively, and the protein coupling amount was 10,000 RU.

After purification, pure AMPK protein was used for SPR analysis for GENI, PF-06409577 was used as a positive control, and the co-crystal structure with AMPK has been reported [23]. The K*_D_* value of PF-06409577 was 7.19 μM, and the K*_D_* value of GENI was 1.07 μM, showing a stronger binding affinity than that of the positive control. (Figure 5a,b) The analysis of Ka and Kd values indicated that the binding rate of PF-06409577 with AMPK was slower than that of GENI, and the dissociation rates were similar. The binding mode of GENI and AMPK belonged to the “fast up–slow down” binding dissociation mode, which helped understand the metabolic characteristics of drugs in vivo and had a certain guiding effect on drug development. Collectively, these results suggested that GENI could directly bind to AMPK.

The non-covalent interaction between drugs and proteins is an important way to exert drug efficacy, and its mode of action and binding affinity usually determine the utilization and biological activity of drugs. AMPK is a heterotrimer complex consisting of an α subunit containing the kinase domain and two regulatory subunits (β- and γ-) [24]. Different action sites have varied or even opposite pharmacological effects. The molecular mechanism of interaction between the protein and molecule should be investigated thoroughly. After co-incubation of GENI and AMPK, LC-MS/MS measurements were performed to predict the binding sites. As shown in Extended Data Figure S2, ASP148, ASP157 and ASP166 of the AMPK α subunits and LYS148, ASP156, LYS309, and ASP316 of the AMPK γ subunit were possible to the binding sites of GENI. To confirm these results, we designed and customized point mutant plasmids at the seven abovementioned amino acid sites, overexpressed and purified AMPK mutant proteins, and further confirmed the binding of GENI by SPR analysis. No significant dose-dependent response was found after replacing D148, D157, and D166 on the AMPK α subunit with A148, A157, and A166, respectively, and the Kd value showed a faster dissociation rate compared with the AMPK of the wild-type. The binding affinity of AMPK and GENI was significantly affected (Figure 5c–e). After point mutation at the four sites of the AMPK γ subunit, the dose-dependent relationship was lost for replacing K148 and D156, the response (RU) was significantly reduced, and the dissociation rate was significantly accelerated for K309 and D316 (Figure 5f–i). Thus, all of these seven sites affected the binding of GENI and AMPK to varying degrees, and they were located in the C lobe kinase domain of the AMPK α subunit, and the CBS2 and CBS4 domain of the AMPK γ subunit, respectively.

AMPK is an important initiation protein for autophagy induction [25]. In various neurodegenerative diseases, AMPK is involved in neuronal autophagy and plays a crucial role in the degradation of toxic proteins such as Aβ protein aggregates [26]. Subsequently, based on AMPK as the target protein, the downstream of the AMPK signaling pathway of GENI-induced autophagy was investigated in PC12 cells. The autophagy biomarker, LC3B protein, was first examined to confirm the occurrence of autophagy. Then, the autophagy-related signaling pathways were explored, and GENI induced autophagy through the AMPK/mTOR/ULK1/LC3B signaling pathway (Extended Data Figure S3a,b, Supplementary Figure S7). Furthermore, the changes in specific proteins related to the autophagy signaling pathway in the hippocampus and cerebral cortex of mice were determined. The results indicated that the AMPK/mTOR/ULK1/LC3B signaling pathway was involved in the anti-AD of GENI (Extended Data Figure S3c–f, Supplementary Figure S8).

The safety of compounds is particularly important in the development of new drugs. At the end of the animal experiment, the epididymis fat, heart, liver, spleen, and kidney of the mice were removed for weighing analysis. There were no significant differences between the mice in each group, as shown in Supplementary Table S1. Meanwhile, the biochemical indicators of blood, such as triglyceride (TG), glucose (GLU), aspartate aminotransferase (AST), alanine aminotransferase (ALT), direct bilirubin (DBIL), total bilirubin (TBIL) and albumin (ALB), were measured and shown in Supplementary Table S2. The plasma TG of mice in the GENI administration groups significantly decreased at doses of 25 and 50 mg/kg (*p* < 0.001, *p* < 0.001). In addition, there were no significant differences in other indexes in the GENI administration group compared with the D-gal-induced group, indicating that GENI at the dose range of 12.5–50 mg/kg was safe and effective and had no toxic side effects.

### 3.5. AMPK as an Anti-AD Drug Target Protein of GENI Was Validated in Natural Aging Mice with Memory Dysfunction

**Figure 6:**
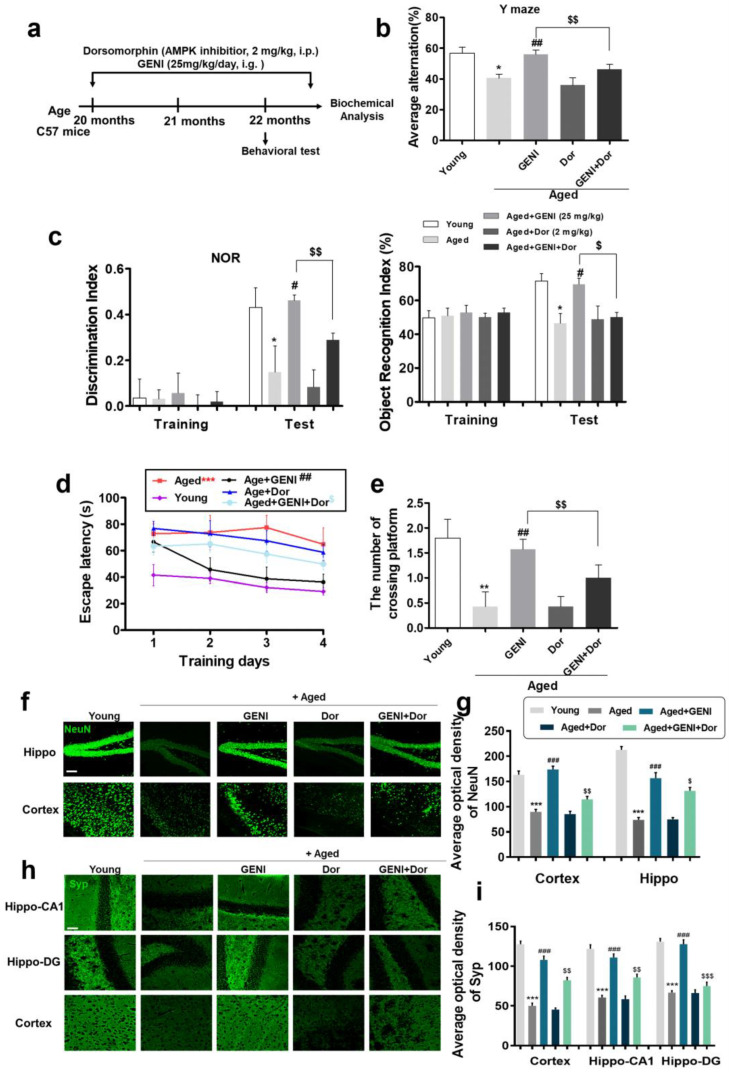
Effect of AMPK inhibitor on the anti-AD effect of GENI in natural aging AD mice. (**a**) Scheme of animal experiment. (**b**) Average alternation of mice in the Y-maze experiment. (**c**) Changes in the discrimination index and recognition index of mice in the NOR test. (**d**) Changes in escape latency of mice in each group on training days 1–4 in water maze test. (**e**) Number of crossing platforms of mice in each group on test day 5 in the water maze test. (**f**,**g**) Changes in the neurons in the hippocampus and cortex of the brain in mice after treating GENI and GENI plus Dor. (**h**,**i**) Effect of GENI and GENI plus Dor on Syp in the hippocampus CA1, DG and cortex. Scale bar, 100 μm. In the behavior test, the animal number was 8. In immunohistochemical staining, n = 7 pictures from 2 mice. Data were presented as mean ± SEM. * *p* < 0.05, ** *p* < 0.01, and *** *p* < 0.001 represented a significant difference from the young control group; # *p* < 0.05, ## *p* < 0.01 and ### *p* < 0.001 represented a significant difference from the aged control group. $ *p* < 0.05, $$ *p* < 0.01 and $$$ *p* < 0.001 represented a significant difference from the GENI-treated aged group.

To confirm whether AMPK is the drug target protein, which GENI produced neuroprotection and anti-AD effects, we used Dor, an inhibitor of AMPK, to interfere with the activity of AMPK and check the anti-AD effect of GENI in C57BL/6j aging mice with memory dysfunction. GENI at a dose of 25 mg/kg as the optimal dose was administered intragastrically once a day, whereas Dor at a dose of 2 mg/kg was injected intraperitoneally once every 2 days for 10 weeks (Figure 6a). Animal behavioral experiments including Y maze, NOR, and water maze tests were performed to evaluate the memory change in AD mice after giving GENI and Dor for eight weeks. The results showed that the improvement in cognitive function, such as alternating arm entry rate, and novel object recognition index of AD mice by GENI in Y maze and NOR tests, significantly decreased due to Dor. (Figure 6b,c, *p* < 0.01, *p* < 0.01 for Y maze test; *p* < 0.05, *p* < 0.01 for NOR test), respectively. During the water maze test, GENI could significantly reduce the escape latency of AD mice during the first 4 days of the training phase (Figure 6d, *p* < 0.01) and increase the number of crossing platforms in the GENI group on the fifth day of the test phase day (Figure 6e, *p* < 0.01). However, these parameters were partially reduced by Dor (Figure 6d,e, *p* < 0.05, *p* < 0.01). Furthermore, we detected the pathological changes in the cerebral cortex and hippocampus of the brain in aging mice via the immunohistochemical technique. The increase in neurons and synaptophysin expression in the hippocampus and cortex of brain in aging mice by GENI were significantly diminished by the Dor (Figure 6f,g, *p* < 0.001, *p* < 0.001 and *p* < 0.01 for neuron in cortex; *p* < 0.001, *p* < 0.001 and *p* < 0.05 for neuron in hippocampus; Figure 6h,i, *p* < 0.001, *p* < 0.001 and *p* < 0.01 for synaptophysin in cortex; *p* < 0.001, *p* < 0.001 and *p* < 0.01 for synaptophysin in hippo-CA1 region; *p* < 0.001, *p* < 0.001 and *p* < 0.001 for synaptophysin in hippo-DG region). Meanwhile, the changes in APP, Tau in hippocampus CA, DG, and cerebral cortex, Aβ, and Tau phosphorylation of cerebral cortex were also detected, respectively (Extended Data Figure S4). The APP and Tau of hippocampus CA, DG, and cerebral cortex, Aβ and Tau phosphorylation of cerebral cortex in the aged group were significantly increased compared with the young group (Extended Data Figure S4a–g and Supplementary Figure S9, *p* < 0.001, *p* < 0.001, *p* < 0.001, *p* < 0.001, *p* < 0.001, *p* < 0.001, *p* < 0.01, *p* < 0.001, *p* < 0.001). After administrating GENI, these proteins were significantly decreased compared with aged group as well as the effects of GENI on these proteins were inhibited by Dor (Extended Data Figure S4a–g, *p* < 0.001, *p* < 0.05, *p* < 0.001 for APP; *p* < 0.01, *p* < 0.001, *p* < 0.001 for Tau; *p* < 0.01 for Aβ oligomers; *p* < 0.05 for *p*-Tau monomers, *p* < 0.01 for p-Tau oligomers). In addition, the anti-inflammation effects of GENI on activated microglia and astrocytes and iNOS expression were significantly suppressed by Dor (Extended Data Figure S5a–d, *p* < 0.001, *p* < 0.001 and *p* < 0.01). Furthermore, we also checked the change in the activity of specific proteins located in the AMPK/mTOR/ULK1 signaling pathway in the hippocampus and cortex of the AD mice (Extended Data Figure S5e–h, Supplementary Figure S10). The activation of this signaling pathway by GENI in the hippocampus and cortex was suppressed by Dor. In conclusion, GENI exerted its neuroprotective effect by directly targeting AMPK, resulting in anti-AD effects.

## 4. Discussion

GENI is an anti-aging leading compound discovered in our previous research [14]. In the present study, our focus is on the investigation of the anti-AD effects, and mechanism of action of GENI. Since the pathogenesis of AD is not well understood, developing the drugs to treat AD is a challenging task. Based on the anti-aging activity of GENI, we utilized two different kinds of AD models: D-gal-induced AD mice and naturally aged mice with AD, which mimic the conditions more similar to the human AD to evaluate the anti-AD effects of this molecule. The results of PC12 cells and animal behavior tests in Figure 1b, Figure 2b–h, and Figure 6b–e and Extended Data Figures S4 and S5 indicated that GENI has neuroprotection and anti-AD effect. The optimal dose for GENI’s anti-AD effect was found to be 25 mg/kg, which was comparable to the effect of DON at a dose of 3 mg/kg. At this point, our results were consistent with another report [27]. The geniposidic acid (GPA) which has the same parent nucleus as GENI, could significantly improve spatial learning and memory abilities in APP/PS1 mice, and reduce Aβ deposition in their brains. GPA not only inhibited the activation of astrocytes and microglia, downregulated IL-1β, IL-6, TNF -α, and iNOS, and upregulated the expression of IL-4 and Arg-1, but also downregulated the gene expression of high mobility group box-1 (HMGB-1) receptor TLR4, and then mediate MyD88, TNF receptor-related factor 6, and phosphorylated ERK1/2, regulating the expression of NF-κB family members.

Oxidative stress and autophagy played important roles during the formation of AD [28]. ROS is the cause of mitochondrial dysfunction, and directly affects neuronal synaptic activity and neurotransmission, leading to cognitive impairment. Autophagy is involved in the degradation of dysfunctional proteins, and a reduction in autophagy leads to the deposition of harmful proteins that trigger neurodegenerative diseases [29]. To understand whether and how GENI regulated oxidative stress and autophagy to exhibit neuroprotection and anti-AD effect, we detected the biomarkers related to oxidative stress, anti-oxidative stress, autophagy, and the expression changes in specific proteins located in signaling pathway at both cell and animal levels. The changes in ROS, MDA, and autophagical flux in Figure 1b–i, reduction in phosphorylate-mTOR, and increase in phosphorylate-AMPK, phosphorylate-ULK1 and LC3B at protein level after giving GENI in Extended Data Figure S3a–f clarified that GENI shows neuroprotection and anti-AD effects through anti-oxidative stress and autophagy induction via AMPK/mTOR/ULK1/LC3B signaling pathway. Moreover, inflammation is also one of the important factors to lead AD [30]. Therefore, we consider that suppressing the inflammation caused by excessive activation of microglia might be one of the most effective strategies in the treatment of AD [31]. Therefore, we also investigated the inflammation parameters at cell and animal levels after treatment of GENI. The changes in pro-inflammatory cytokines in Extended Data Figure S1a–d, and the activity of microglia and astrocytes in the cerebral cortex and hippocampus in Figure 3a,d–f confirmed that anti-inflammation is also involved in the neuroprotection and anti-AD effects of GENI.

The AMPK signaling pathway takes part in the occurrence and development of AD by regulating oxidative stress and mitochondrial function, and the GSK3β, a downstream protein of the AMPK signaling pathway is pathologically correlated with Aβ in AD [32]. In addition, nuclear factor erythroid 2-related factor 2 (Nrf2) is a critical transcription factor that regulates the body’s anti-oxidative stress. Some evidence indicated that Nrf2 activation can delay cognitive dysfunction in various AD models by affecting mitochondrial function [33]. Notably, the expression level of GSK3β was elevated in Nrf2 knockdown tissues and cells, while inhibition of GSK3β led to Nrf2 activation under oxidative stress in AD [34]. Therefore, targeting AMPK/GSK3β/Nrf2, as well as AMPK/NRF2 signaling and AMPK/GSK3β/Nrf2 pathways balance might be a promising therapeutic strategy. Furthermore, Nrf2 regulates redox homeostasis and plays a role as an anti-inflammatory factor in various inflammatory-degenerative disorders [35]. The redox-active compounds of natural products acting via hormetic dose responses, are endowed with powerful anti-inflammatory effects [36]. Thus, the interplay and coordination of redox interactions and their interaction with endogenous and exogenous antioxidant defense systems is an emerging area of research interest in anti-inflammatory and anti-degenerative therapeutics.

The hippocampus and cerebral cortex of the brain are key regions for memory. The hippocampus essentially worked for memory formation and storage of short-term memory traces, while the cerebral cortex participated in long-term storage of memory. Furthermore, the hippocampus plays a crucial role in the transfer of memory traces to the cerebral cortex, facilitating memory consolidation and long-term storage, and engages in feedback interactions that are essential for memory retrieval [37]. Considering that AD is associated with a variety of neuronal and synaptic dysfunctions [38], we detected the changes in neurons and synapses in the cerebral cortex and hippocampus with immunohistochemical staining. The significant increase in mature neurons in Figure 3a,b, synaptophysin in Figure 3a–c, reducing pro-inflammatory cytokines in Figure 3a–d, and weakening glial cell activation in Figure 3a,e,f clarified that GENI improved memory and cognitive impairment in AD mice via neuroprotection.

Iridoid glycoside is a class of natural products that widely exist in nature, and it possesses a wide range of pharmacological activities. However, a target identification study of iridoid glycosides corresponding to different biological activities is lacking, especially the drug target of iridoid glycosides against AD. According to the literature [22], and based on the results of the induction of autophagy by GENI, we conducted target screening by applying DARTS and CETSA methods on several recognized targets of autophagy to discover the candidates of target protein for GENI. The results in Figure 4a–h estimated that AMPK was the potential target protein of GENI. To further confirm the possibility of AMPK as the target protein of GENI, we performed inhibitor assay, siRNA interference experiments, and SPR analysis. The results of these experiments in Figure 4i–n and Figure 5a,b suggested that AMPK was a target protein of GENI. To further understand the binding sites of AMPK and GENI, we conducted LC-MS/MS analysis, mutants of AMPK, and SPR analysis. The results in Figure 5c–i confirmed that GENI exhibited stronger binding affinity than the positive molecule PF-06409577. The binding sites of GENI and AMPK were ASP148, ASP157, and ASP166 in the C lobe kinase domain of the AMPK α subunit, LYS148 and ASP156 in the CBS2 domain, and LYS309 and ASP316 in the CBS4 domain of the AMPK γ subunit. Regrettably, we failed to obtain the crystal of AMPK and determine the three-dimensional structure of GENI binding to AMPK.

As a central molecular sensor, AMPK plays a key role in maintaining cellular energy homeostasis. Specific AMPK silencing could affect energy metabolism and synaptic plasticity in the mouse hippocampus, ultimately impacting cognitive function [39]. In addition, AMPK played an important role in the Aβ deposition and phosphorylation tau in AD [40]. Specifically, AMPK acted as a connector for several important signaling pathways that control protein synthesis, autophagy, and lipid metabolism. Dysregulation of these processes can contribute to the development and progression of AD [41]. To confirm whether AMPK is the pharmacodynamics target of GENI for AD treatment, we detected the anti-AD effect again after we used the AMPK inhibitor, Dor to interfere AMPK function of the brain in the natural-aged AD mice. The results of animal behavior experiments in Figure 6 and Extended Data Figures S4 and S5 showed that AMPK was the drug target protein for the anti-AD effect of GENI. Since we selected the natural-aged mice with 20 months as AD mouse model, their physiological state was very weak. To avoid fatal damage to them, we used the half-dose of Dor as reported to perform the experiment. Considering the possibility that Dor cannot completely diminish the anti-AD effect of GENI, we plan to use a gene edit technique, such as shRNA of AMPK to knock down the AMPK of the hippocampus and cerebral cortex to evaluate the anti-AD effect of this small molecule in the future study.

AMPK is a heterotrimeric protein that contains α, β, and γ subunits. Among them, α subunit serves as a catalytic role, while β and γ subunits play an important role in maintaining trimer stability and substrate specificity [13]. AMPK has two main forms of activity regulation: firstly, it can be activated by a series of upstream kinases, such as LKB1, CAMKK2, TAK1, and AMPK, all of which phosphorylate AMPK α subunit at threonine 172 [3,42]. Similarly, the activity of AMPK is also regulated by the AMP/ATP ratio in cells. Recently, the compounds that directly or indirectly promote AMPK activity have been developed, and their action modes have been clarified. Some of them have been approved for marketing or are currently undergoing clinical trials. The direct agonists of AMPK, such as salicylate which acts on the subunits of AMPK to exert their effects, can cause conformational activation of AMPK [43]. Additionally, compounds like GSK621 enhance the phosphorylation level of AMPK while maintaining its protein kinase activity [44]. On the other hand, as indirect agonists of AMPK, PT-1 and metformin activate AMPK by inhibiting mitochondrial electron transfer chains or inducing mitochondrial uncoupling [45], thereby reducing ATP synthesis, while amarogentin and RSVA 405 activate AMPK upstream kinases [46]. Although some AMPK agonists have entered the clinical development stage, there are still some problems, such as the possibility that activation of AMPK may promote myocardial hypertrophy or enhance the survival ability of cancer cells under hypoxic conditions, which require more extensive clinical trials to determine its safety and efficacy [19,47,48]. In our study, we found that GENI, as an AMPK activator, activates AMPK to produce an anti-AD effect by directly binding with the C lobe kinase domain of α and CBS2 and CBS4 domain of γ subunits. Moreover, we did not find the myocardial hypertrophy and toxic side effects after giving GENI (Supplementary Tables S1 and S2). Therefore, GENI is considered a potential candidate for a drug molecule for anti-AD drug development.

## 5. Conclusions

Taken together, this study demonstrated that GENI directly activated AMPK to improve the memory dysfunction of AD mice via inducing autophagy, anti-oxidative stress, and anti-neuroinflammation through AMPK/mTOR/ULK1/LC3B signaling pathway. The binding sites of GENI and AMPK provided important information for further structural optimization of GENI. These findings propose a new way to directly activate AMPK to play a neuroprotective role in the treatment of AD.

## Data Availability

All figures and data used to support this study are included in this article.

## Supplementary Materials

The following supporting information can be downloaded at: https://www.mdpi.com/article/10.3390/antiox14010057/s1, Extend data Figure S1: Anti-neuroinflammation effect of GENI on BV-2 cel; Extend data Figure S2: LC-MS/MS analysis for the binding site of GENI and AMPK; Extend Data Figure S3: Interaction of between AMPK and GENI, and binding sites confirmation of GENI with wild-type and mutants of AMPK by SPR analysis; Extend data Figure S4: The effect of AMPK inhibitor Dor on the anti-neuroinflammatory and autophagy effects of GENI at the animal level; Supplementary Table S1: Changes in organs weight in each group in the D-gal induced AD mice experiment; Supplementary Table S2: Effects of GENI on serum biochemical index of D-galactose mice; Supplementary Table S3: List of primers sequence in RT-PCR; Supplementary Table S4: List of antibodies used in the Western blot analysis; Supplementary Table S5: List of antibodies used in the immunohistochemistry; Supplementary Figure S1: The original data of Western blot analysis of Figure 1m,n; Supplementary Figures S2: The original data of Western blot analysis of Figure 4a,b; Supplementary Figure S3: The original data of Western blot analysis of Figure 4c–e; Supplementary Figure S4: The original data of Western blot analysis of Figure 4f–h; Supplementary Figure S5: The original data of Western blot analysis of Figure 4i,k; Supplementary Figure S6: The original data of Western blot analysis of Figure 4l–o; Supplementary Figure S7: The original data of Western blot analysis of Extend Data Figures S3a,b; Supplementary Figure S8: The original data of Western blot analysis of Extend Data Figure S3c,f; Supplementary Figure S9: The original data of Western blot analysis of Extend Data Figure S4e,f. Supplementary Figure S10: The original data of Western blot analysis of Extend Data Figure S5e,g.

## Abbreviations

Aβ	Amyloid beta
AD	Alzheimer’s Disease
CETSA	Cellular thermal shift assay
CQ	Chloroquine
DARTS	Drug affinity responsive target stability
DBIL	Direct bilirubin
DON	Donepezil
D-gal	D-galactose
Dor	Dorsomorphin
GPA	Geniposidic acid
GENI	Geniposidic 4-isoamyl ester
GLU	Glucose
HMGB-1	High mobility group box-1
MTT	3-(4,5-dimethyltaizol-2-yl)-2,5-diphenyltetrazolium bromide
MWM	Morris water maze
iNOS	Inducible nitric oxide synthase
IL-6	Interleukin-6
NOR	Novel object recognition
Rap	Rapamycin
RES	Resveratrol
ROS	Reactive oxygen species
PET	Positron emission tomography
PI3K	Phosphatidylinositol-3 kinase
SPR	Surface plasmon resonance
Syp	Synaptophysin
