# Supplementary material for: GENI as an AMPK Activator Binds α and γ Subunits and Improves the Memory Dysfunction of Alzheimer’s Disease Mouse Models via Autophagy and Neuroprotection
Source: Antioxidants (Basel). 2025 Jan 6;14(1):57. doi: 10.3390/antiox14010057 (PMC11763059; doi:10.3390/antiox14010057)
Supplement: Supplementary file 1 [file antioxidants-14-00057-s001.zip › antioxidants-3380498-supplementary.pdf]

Supplementary information

GENI as an AMPK activator binds α and γ subunits and improves the memory dysfunction of Alzheimer's disease mouse models via autophagy and neuroprotection

Ying Wang [1,#], Lanjie Li [1,#], Danni Chen [1], Jiaheng Shan [1], Meijuan Yi [1], Hiroyuki Osada [2], Minoru Yoshida [3], Lan Xiang [1,*], and Jianhua Qi [1,4,*]

[1] College of Pharmaceutical Sciences, Zhejiang University, Yu Hang Tang Road 866, Hangzhou 310058, China; 11819026@zju.edu.cn (Y.W.); lilanjie@zju.edu.cn (L.L.); 12019025@zju.edu.cn (D.C.); 22119073@zju.edu.cn (J.S.); 22119153@zju.edu.cn (M.Y.)
[2] Chemical Biology Research Group, RIKEN Center for Sustainable Resource Science, Wako-shi,
Saitama 351-0198, Japan; osadahiro@riken.jp
[3] Chemical Genomics Research Group, RIKEN Center for Sustainable Resource Science, Wako,
Saitama 351-0198, Japan; yoshidam@riken.jp
[4] Jinhua Institute, Zhejiang University, Jinhua 321299, China
* Correspondence: lxiang@zju.edu.cn (L.X.); qijianhua@zju.edu.cn (J.Q.); Tel.: +86-571-88208627 (J.Q.)
† These authors contributed equally to this work.

1. Material and methods

1.1. Real-time polymerase chain reaction (RT-PCR) analysis

PC12 cells (2×10^6) were seeded in 60 mm dishes and treated with RES (10 μM) or GENI (3 or 10 μM) for 24 h. Total RNA was extracted with TRIzol Reagent (Beijing Cowin Biotech Company, Beijing, China). RNA content was determined using an Eppendorf Biophotometer Plus (Eppendorf Company, Hamburg, Germany). Transcription was performed using 5 μg of total RNA, Oligo (dT)20 primer and reverse transcriptase (Beijing Cowin Biotech Company, Beijing, China). Transcript levels were quantified by real-time PCR (AB SCIEX, Massachusetts, USA) and SYBR Premix EX Taq™ (Takara, Otsu, Japan). Primers (Sangon Biotech Co. Ltd., Shanghai, China) used in this study are given in Supplementary Table S3. The thermocycling parameters for PC12 cells were as follows: SOD1 and SOD2: 95 °C for 2 min, then 40 cycles, 54 °C for 35 seconds; Bcl-x1 and Nrf2: 95 °C for 2 min, then 40 cycles, 95 °C for 15 seconds, 60 °C

for 15 seconds, 68 °C for 20 seconds. All results were normalized to GAPDH RNA levels, and relative mRNA transcript levels were calculated using the $^{\Delta\Delta Ct}$ formula.

1.2. Enzyme-linked immunosorbent assay

BV-2 cells were treated with LPS (1 μg/mL) in the absence or presence of GENI (1, 3 and 10 μM). The cell supernatant was collected at different time points for proinflammatory mediator assay (8 h for IL-6 and 24 h for NO). NO production was quantified using an NO assay kit (S0021S, Beyotime, China), and IL-6 was detected using commercial ELISA kits (EM004, ExCell, China) according to the manufacturer's instructions.

For NO content evaluation, firstly, the NO assay kit was removed, and Griess Reagents I and II were warmed to room temperature. The standard substance was diluted with cell culture solution to 0, 1, 2, 5, 10, 20, 40, 60 and 100 μM. Secondly, 50 μL of standard reagent and the diluted sample were added into a 96-well plate. A 50 μL mixture of Griess Reagent I and Griess reagent II was added into each well, which contained standard reagent or sample. The absorbance was determined with a microplate reader (BioTek, California, USA) at 540 nm.

For IL-6 content evaluation, we prepared samples, standards and biotinylated antibody working fluid in advance. The standard product gradient was diluted to 1000, 500, 250, 125, 62.5, 31.25, 15.625 and 0 pg/mL for use, and blank holes were set. The 100 μL sample or standard substance of different concentrations was added to the corresponding hole, and about 50 μL of biotinylated antibody working liquid was added to the sample and standard substance hole. The reaction hole was sealed with sealing plate adhesive paper. The working liquid of the enzyme conjugate was prepared 30 min in advance by incubating at room temperature with a 300 rpm micro-oscillator for 120 min. The sample was placed at room temperature away from light, and the board was washed five times after incubation. In addition to the blank holes, 100 μL of enzyme conjugate working liquid was added to each hole, and the reaction holes were sealed with plate adhesive paper. The board was incubated at room temperature with a 300 rpm micro-oscillator for 60 min and then washed five times. We added 100 μL of chromogenic substrate to each well and incubated the samples at room temperature for 15 min away from light. About 100 μL of termination solution was added to each hole, and we measured the OD_{450} value immediately after mixing with a microplate reader (BioTek, California, USA).

1.3. Western blot analysis.

For PC12 cell samples, we seeded 2 × 10^6 PC12 cells in each 60 mm dish containing 5 mL of DMEM and incubated the dish for 24 h. For the dose-dependent experiment of GENI, GENI (1, 3 and 10 μM) was added to the dishes and incubated for a period of time (2 hours for p-AMPK α, AMPK α, 48 hours for p-mTOR, mTOR and p-ULK1, ULK1; 8 hours for LC3-I/II). Cells were then collected, lysed using RAPI lysis buffer and centrifuged. The supernatants of each group were used as protein samples. For the protein samples of autophagy flow assay, we seeded 2 × 10^6 PC12 cells in each 60 mm dish containing 5 mL of DMEM and incubated the dish for 24 h. The GENI, rapamycin and inhibitors, such as chloroquine and 3-MA were added according to the method of autophagy flow assay. After treatment of 18 h, cells were then collected, lysed using RAPI lysis buffer and centrifuged. The supernatants of each group were used as protein samples.For the protein samples of autophagy flow assay, we seeded 2 × 10^6 PC12 cells in each 60 mm dish containing 5 mL of DMEM and incubated the dish for 24 h. The GENI, rapamycin and inhibitors, such as chloroquine and 3-MA were added according to the method of autophagy flow assay. After treatment of 18 h, cells were then collected, lysed using RAPI lysis buffer and centrifuged. The supernatants of each group were used as protein samples. For animal samples, an appropriate amount of tissue, such as cerebral cortex and hippocampus (50–100 mg), of each group was weighed, and we added lysis buffer with 1:9 ratios. After grinding and centrifuging, the supernatants of each group were used as protein samples. The protein concentration was measured with a BCA assay kit (Beijing Cowin Biotech Company, Beijing, China). Sodium dodecyl sulphate polyacrylamide gel electrophoresis (SDS-PAGE) was used to separate proteins (15 μg), which were transferred to PVDF membranes. The membrane was incubated with primary and secondary antibodies (Supplementary Table S5 for details). Antigens were visualised using a high-sensitivity chemiluminescence detection kit (Beijing Cowin Biotech Company, Beijing, China). Bands were quantitatively measured using ImageJ software (National Institutes of Health, Bethesda, MD, USA).

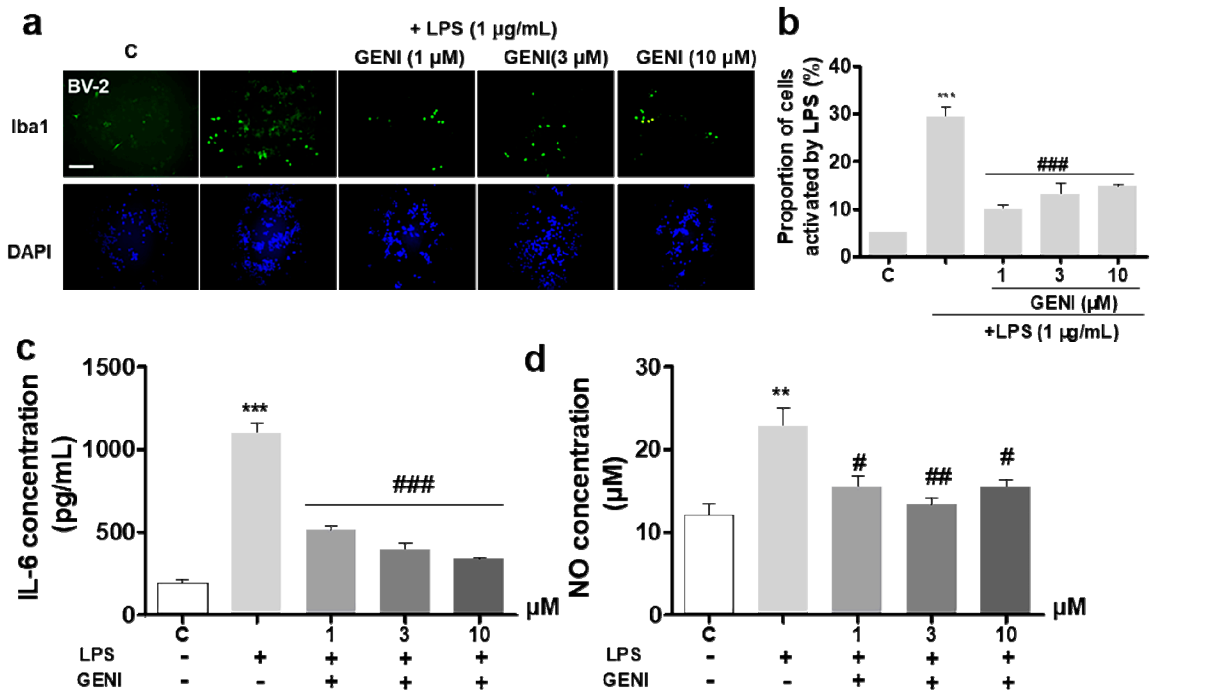

Extended Data Figure S1 Anti-neuroinflammation effect of GENI on BV-2 cell. (a-b) Photomicrograph of microglia activated by lipopolysaccharide (LPS). BV-2 cells were pretreated with 1 μg/mL LPS to evaluate the activity of GENI at different concentrations on activated microglia. Scale bar, 30 μm. (**c-d**) Effect of GENI on the levels of inflammatory factors IL-6 and NO in BV-2 cell culture supernatant. Activation of BV-2 cells was induced with LPS (1 μg/mL). The experiment was repeated three times, $^{**}p < 0.01$ and $^{***}p < 0.001$ represented a significant difference compared with the negative control group, $^{\#}p < 0.05$, $^{\#\#}p < 0.01$ and $^{\#\#\#}p < 0.001$ represented a significant difference compared with the LPS pretreatment group.

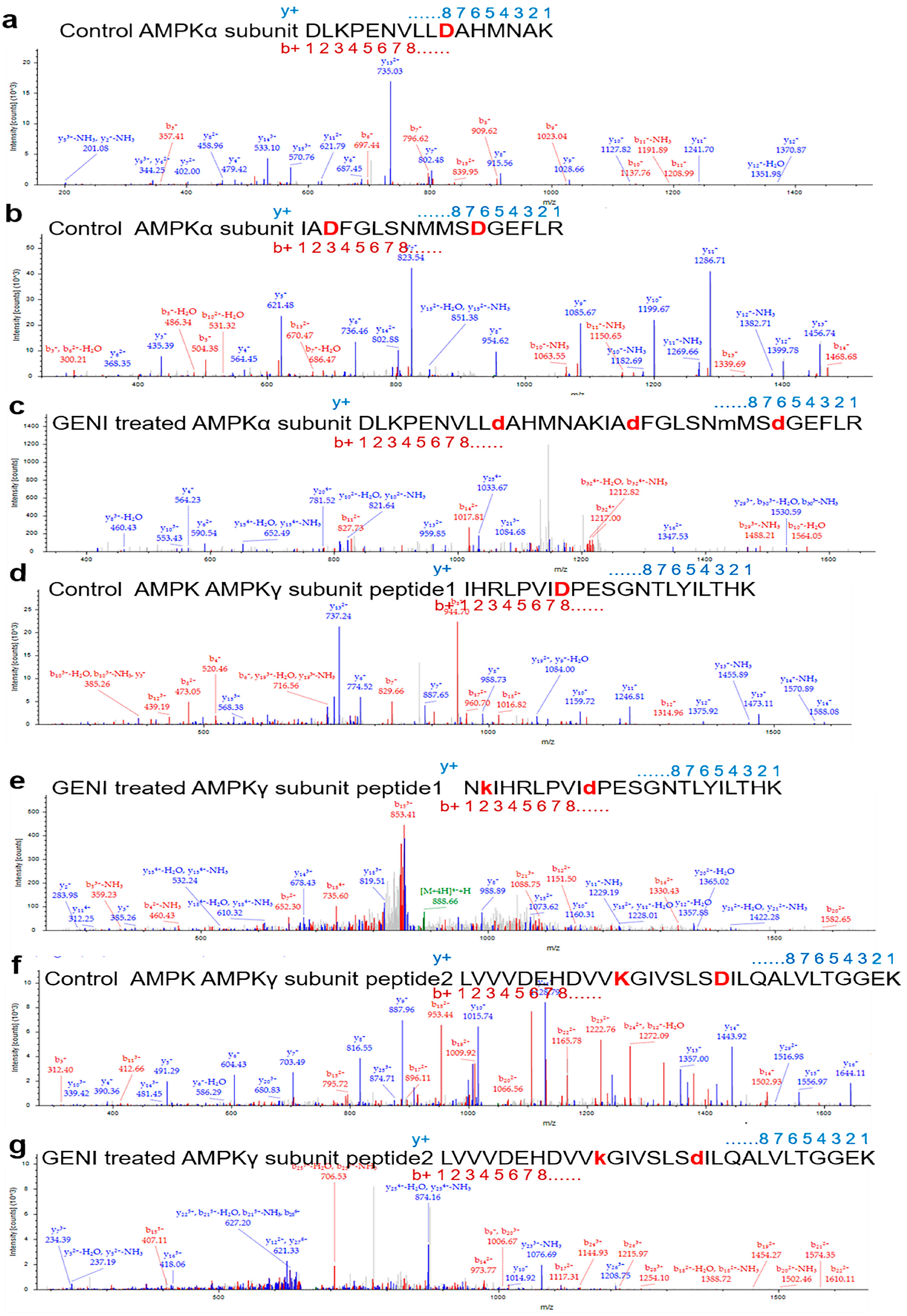
a
Control AMPKα subunit DLKPENVLLDAHMNAK
b
Control AMPKα subunit IADFGLSNMMSDGEFLR
c
GENI treated AMPKα subunit DLKPENVLLdAHMNAKIAdFGLSNmMSdGEFLR
d
Control AMPK AMPKγ subunit peptide1 IHRLPVIDPESGNTLYILTHK
e
GENI treated AMPKγ subunit peptide1 NkIHRLPVIdPESGNTLYILTHK
f
Control AMPK AMPKγ subunit peptide2 LVVVDEHDVVKGIVSLSDILQALVLTGGEK
g
GENI treated AMPKγ subunit peptide2 LVVVDEHDVVkGIVSLSdILQALVLTGGEK
y+ 8 7 6 5 4 3 2 1
b+ 1 2 3 4 5 6 7 8......
m/z

Extended Data Figure S2 LC-MS/MS analysis for the binding site of GENI and AMPK. (**a-c**) GENI binds to ASP148, ASP157 and ASP166 of AMPKα subunit. (**d-g**) GENI binds to LYS148, ASP156, LYS309 and ASP316 in AMPKγ subunit.

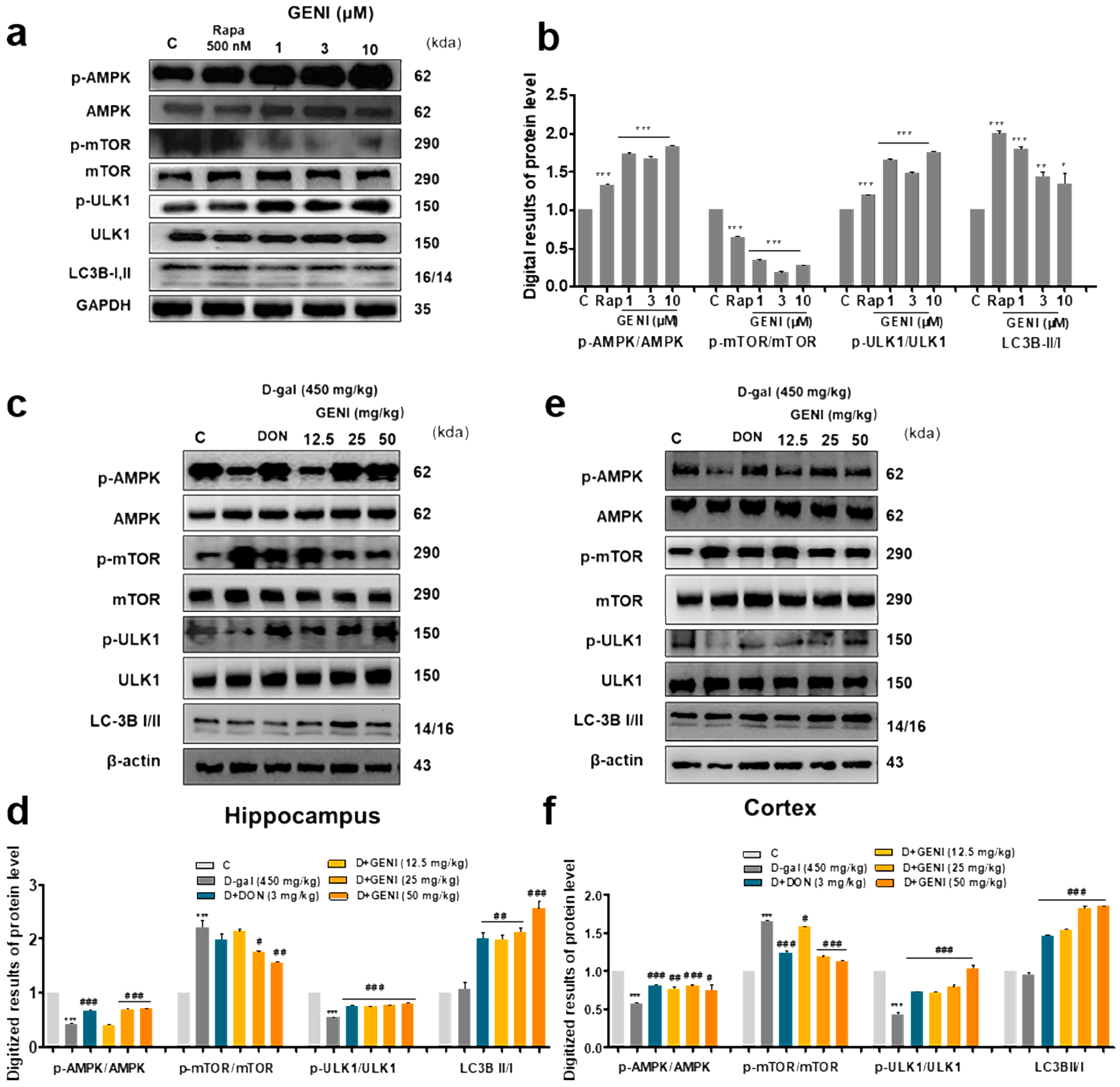

Extended Data Figure S3 Exploration of signaling pathways of GENI.

(**a** and **b**) Western blot analysis for specific proteins in the autophagy signaling pathway in PC12 cells. Rapamycin (Rap) at a dose of 0.5 μM was used as a positive control. The repeat number is three independent experiments. Data were presented as mean ± SEM. *p<0.05, **p<0.01 and ***p<0.001 represented a significant difference from the blank control group. (**c**–**f**) Western blot analysis for specific proteins in autophagy signaling pathway in hippocampus (**c** and **d**) and

cortex (**e** and **f**). The samples of five mice in each group were mixed. Data are presented as mean ± SEM. ***p < 0.001 represented a significant difference from the control group, #p < 0.05, ##p < 0.01 and ###p < 0.001 represented a significant difference from the D-gal group.

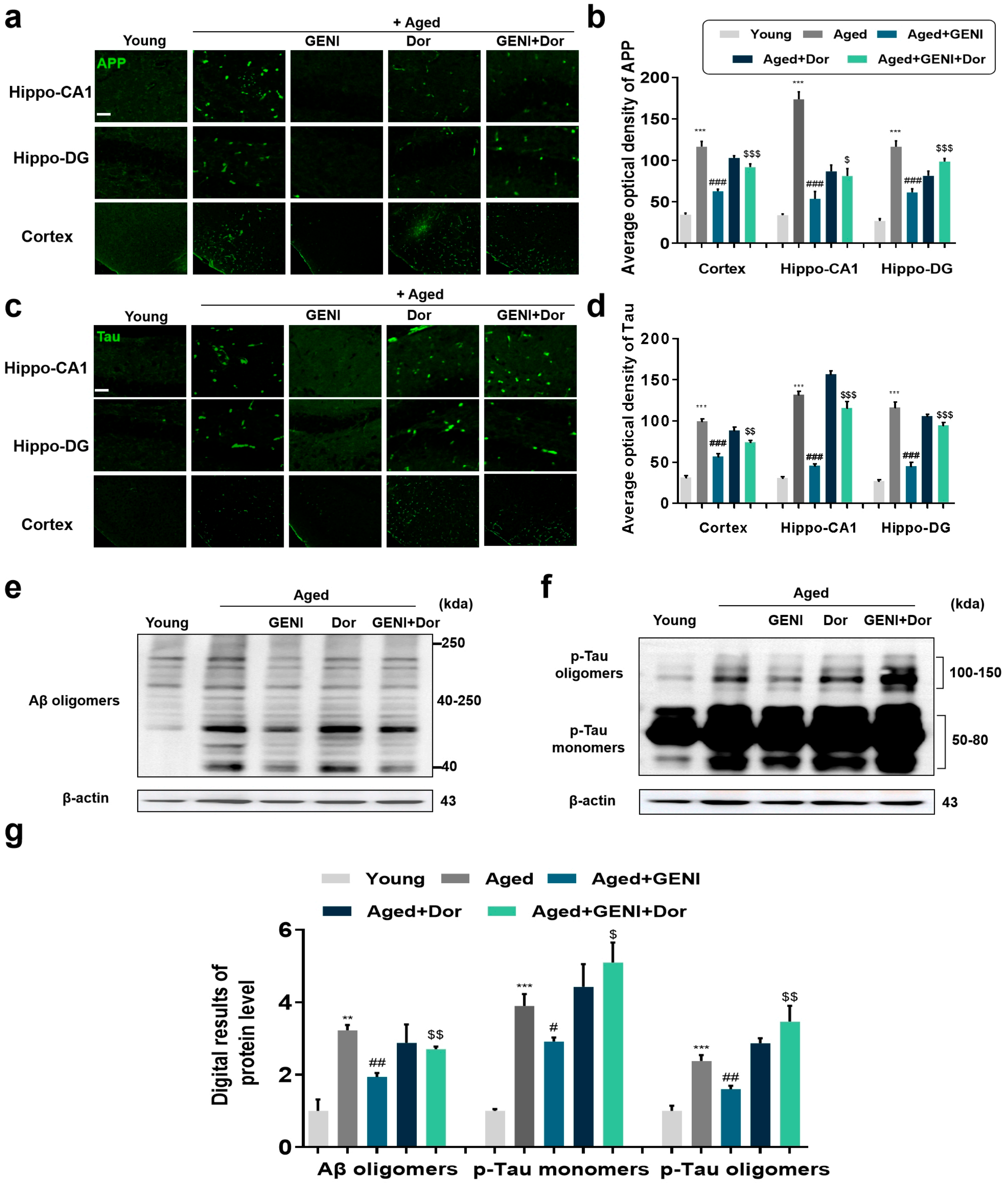

a
Young
+ Aged
GENI
Dor
GENI+Dor
APP
Hippo-CA1
Hippo-DG
Cortex
b
Young
Aged
Aged+GENI
Aged+Dor
Aged+GENI+Dor
Average optical density of APP
Cortex
Hippo-CA1
Hippo-DG
c
Tau
d
Average optical density of Tau
e
Aged
(kda)
Aβ oligomers
β-actin
250
40-250
40
43
f
p-Tau oligomers
p-Tau monomers
100-150
50-80
g
Digital results of protein level
Aβ oligomers
p-Tau monomers
p-Tau oligomers

Extended Data Figure S4 The AMPK inhibitor Dor inhibited the effects of GENI on the APP, Tau, Aβ and phosphorylation Tau of brain at the animal level. (a-d) The results of APP and Tau (green) staining in hippocampal CA1, DG, and cortex in the mouse brain, as assessed by average optical density. Two mice were used in each group and six photographs were collected. Scale bar, 60 μm. ***p < 0.001 represented a significant difference compared with the younger group; ###p < 0.001 represented a significant difference compared with the aging group; $p <0.05, $$p < 0.01, $$$p < 0.001 represented a significant difference compared to the GENI-administered group of aged AD mice. (e-g) The western blotting results of Aβ and phosphorylation Tau of cerebral cortex in aged mice after giving GENI and AMPK inhibitor, Dor. Equal numbers of 5 mice in each group were sampled, mixed and loaded. *p < 0.05, **p < 0.01 and ***p < 0.001 represented a significant difference from the young group; #p < 0.05 and ##p < 0.01 and represented a significant difference compared with the aging group; $p < 0.05 and $$p < 0.01 represented significant differences compared to GENI-administered groups of aging mice.

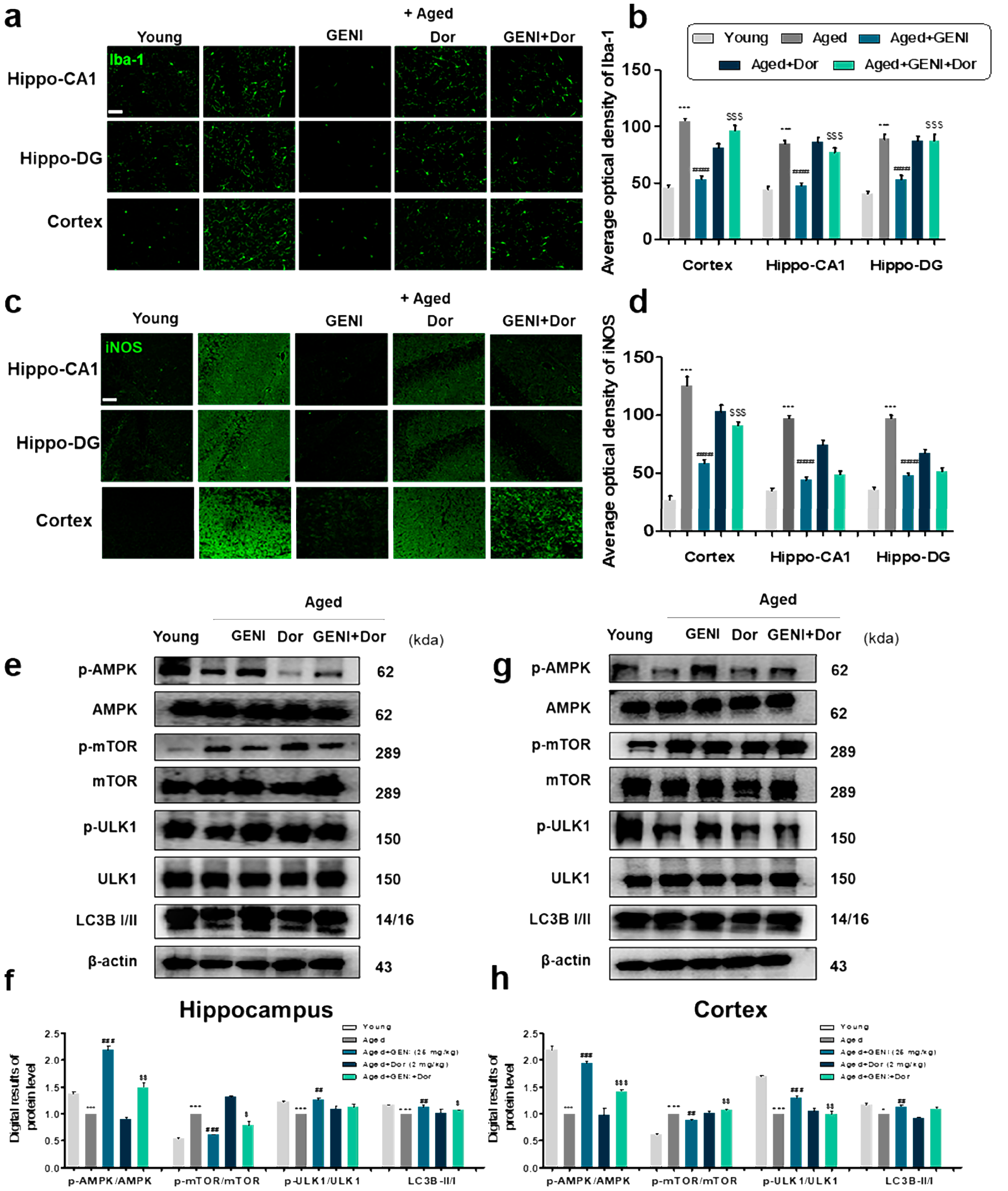

Extended Data Figure S5 The effect of AMPK inhibitor Dor on the anti-neuroinflammatory and autophagy effects of GENI at the animal level. (**a-d**) The results of Iba-1 and iNOS (green) staining in hippocampal CA1, DG, and cortex in the mouse brain, as assessed by average optical density. Two mice were used in each group and six photographs were

collected. Scale bar, 60 μm. ***$p < 0.001$ represented a significant difference compared with the younger group; ###$p < 0.001$ represented a significant difference compared with the aging group; $$$p < 0.001 represented a significant difference compared to the GENI-administered group of aged AD mice. (**e**-**h**) The effect of AMPK inhibitor Dor on the autophagy-related signaling pathway of AMPK/mTOR/ULK1 in the hippocampus and cerebral cortex of mice. Equal numbers of 5 mice in each group were sampled, mixed and loaded. *$p < 0.05$, and ***$p < 0.001$ represented a significant difference from the young group; ##$p < 0.01$ and ### $p < 0.001$ represented a significant difference compared with the aging group; $p < 0.05, $$p < 0.01 and $$$p < 0.001 represented significant differences compared to GENI-administered groups of aging mice.

Supplementary Table S1. Changes of organs weight in each group in the D-gal induced AD mice experiment

Groups	Heart (g)	Liver (g)	Spleen (g)	Kidney (g)	Fat (g)
Control	0.188±0.033	1.573±0.217	0.125±0.029	0.530±0.080	0.687±0.155
D-gal (450 mg/kg)	0.206±0.018	1.578±0.148	0.121±0.006	0.559±0.126	0.721±0.268
D-gal + DON (3 mg/kg)	0.196±0.048	1.568±0.166	0.113±0.015	0.547±0.034	0.728±0.302
D-gal + GENI (12.5 mg/kg)	0.186±0.025	1.583±0.177	0.124±0.026	0.549±0.033	0.731±0.161
D-gal + GENI (25 mg/kg)	0.196±0.043	1.697±0.174	0.109±0.018	0.545±0.068	0.717±0.320
D-gal + GENI (50 mg/kg)	0.172±0.011	1.662±0.216	0.101±0.020	0.546±0.054	0.566±0.118

The animal number was 7;
Data were presented as mean ± SEM;
D-gal represented D-galactose; DON represented Doleperazine; GENI represented geniposidic 4-isoamyl ester.

Supplementary Table S2. Effects of GENI on serum biochemical index of D-galactose mice

Groups	TG (mmol/L)	GLU (mmol/L)	AST (U/L)	ALT (U/L)	DBIL (μmol/L)	TBIL (μmol/L)	ALB (g/L)
Control	0.94±0.21	7.7±0.5	57.1±10.5	23.3±3.7	0.4±0.1	1.9±0.1	25.6±0.7
D-gal (450 mg/kg)	1.08±0.30	7.4±0.4	55.0±14.4	24.0±6.0	0.3±0.1	1.9±0.5	24.0±2.9
D+DON (3 mg/kg)	0.57±0.13###	7.0±0.5	59.0±16.4	24.5±6.5	0.8±0.1###	2.6±0.3#	24.0±1.6
D+GENI (12.5 mg/kg)	0.84±0.10	6.8±0.6	53.3±2.3	23.0±7.7	0.6±0.2	2.3±0.1	23.8±1.7
D+GENI (25 mg/kg)	0.62±0.12###	6.0±0.8	50.6±4.3	25.4±2.4	0.4±0.1	1.8±0.3	24.7±1.6
D+GENI (50 mg/kg)	0.54±0.14###	6.4±0.9	52.2±5.1	25.3±3.6	0.5±0.2	2.1±0.5	24.9±2.1

The animal number was 7;
Data were presented as mean ± SEM;
D-gal represented D-galactose; DON represented Doleperazine; GENI represented geniposidic 4-isoamyl ester;
#$p < 0.05$ and ### $p < 0.001$ represented a significant difference compared with the D-gal group.

Supplementary Table S3. List of primers sequence in RT-PCR

Gene	Species	Sequences
SOD1 (NM_017050.1)	Rat	sense: 5'-AGG GCA TCA TCA ATT TCG AGC-3’
		anti-sense: 5′-ACA TTG CCC AAG TCT CCA AC-3’
SOD2 (NM_017051.2)	Rat	sense: 5'-GGA AGC CAT CAA ACG TGA CT-3′
		anti-sense: 5′-CCT TGC AGT GGA TCC TGA TT-3′
Bcl-x1 (XM_006235265.5)	Rat	sense: 5'-TTC GGG ATG GAG TAA ACT GG-3'
		anti-sense: 5'-TGT CTG GTC ACT TCC GAC TG-3'
Nrf2 (NM_012675.3)	Rat	sense: 5'-TGG TGG TTT GCT ACG ACG-3′
		anti-sense: 5′-CTC CAG AAC TCC AGG CGG-3′
AMPK α1 (XM_063282506.1)	Rat	sense： 5’-ATCCGCAGAGAGATCCAGAA-3'
		anti-sense： 5’-CGTCGACTCTCCTTTTCGTC-3'
AMPK α2 (XM_039110823.2)	Rat	sense： 5’-CGGAGGTCATCTCAGGAAGGCTG-3'
		anti-sense： 5’-ACGTGCTCATCGTCGAACGGG-3'
GAPDH (XM_039107008.2)	Rat	sense: 5'-CAG CCT CGT CTC ATA GAC AAG ATG-3'
		anti-sense: 5′-CAA TGT CCA ACT TTG TCA CAA GAG AAA-3'

Supplementary Table S4. List of antibodies used in the western blot analysis

Primary antibody	Product No.	Tested dilution of primary antibody	Secondary antibody	Tested dilution of Secondary antibody
Phospho-AMPK α-antibody	#2535 Cell Signaling Technology	1:1000	Goat Anti-Rabbit IgG, HRP Conjugated (#CW0103S, Cowin Biotech Company)	1:5000
AMPK α-antibody	#5832 Cell Signaling Technology	1:1000	Goat Anti-Rabbit IgG, HRP Conjugated (#CW0103S, Cowin Biotech Company)	1:5000
Phospho-mTOR-antibody	#2971 Cell Signaling Technology	1:1000	Goat Anti-Rabbit IgG, HRP Conjugated (#CW0103S, Cowin Biotech Company)	1:5000
mTOR-antibody	#2983 Cell Signaling Technology	1:1000	Goat Anti-Rabbit IgG, HRP Conjugated (#CW0103S, Cowin Biotech Company)	1:5000
Phospho-ULK1-antibody	#14202 Cell Signaling Technology	1:1000	Goat Anti-Rabbit IgG, HRP Conjugated (#CW0103S, Cowin Biotech Company)	1:5000
ULK1-antibody	#8054Cell Signaling Technology	1:1000	Goat Anti-Rabbit IgG, HRP Conjugated (#CW0103S, Cowin Biotech Company)	1:5000
LC3B I/II antibody	#2775 Cell Signaling Technology	1:1000	Goat Anti-Rabbit IgG, HRP Conjugated (#CW0103S, Cowin Biotech Company)	1:5000
β-actin Mouse Monoclonal Antibody	#CW0096M, Cowin Biotech Company	1:1000	Goat Anti-Mouse IgG, HRP Conjugated (#CW0102S, Cowin Biotech Company)	1:5000
GAPDH Mouse Monoclonal Antibody	#CW0100, Cowin Biotech Company	1:1000	Goat Anti-Mouse IgG, HRP Conjugated (#CW0102S, Cowin Biotech Company)	1:5000
Aβ antibody	#8243S Cell Signaling Technology	1:1000	Goat Anti-Rabbit IgG, HRP Conjugated (#CW0103S, Cowin Biotech Company)	1:5000
p-Tau (phospho S396) antibody	#ab32057 abcam	1:2000	Goat Anti-Rabbit IgG, HRP Conjugated (#CW0103S, Cowin Biotech Company)	1:5000

Supplementary Table S5. List of antibodies used in the immunohistochemistry

Primary antibody	Product No.	Tested dilution of primary antibody	Secondary antibody	Tested dilution of Secondary antibody
NeuN antibody	#ab177487, Abcam	1:1000	Alexa Fluor-488 conjugated goat anti-rabbit IgG (#ab150077, Abcam)	1:1000
Syp antibody	#ab32127, Abcam	1:500	Alexa Fluor-488 conjugated goat anti-rabbit IgG (#ab150077, Abcam)	1:1000
iNOS antibody	#PA1036, Invitrogen	1:20	Alexa Fluor-488 conjugated goat anti-rabbit IgG (#ab150077, Abcam)	1:1000
GFAP antibody	#AB5541, sigma-Aldrich	1:500	Alexa Fluor-488 conjugated goat anti-chicken IgG (#ab150169, Abcam)	1:1000
Iba-1 antibody	#ab178847, Abcam	1:1000	Alexa Fluor-488 conjugated goat anti-rabbit IgG (#ab150077, Abcam)	1:1000
tau antibody	#66499, proteinintech	1:400	Alexa Fluor-488 conjugated goat anti-mouse IgG (#ab150113, Abcam)	1:500
APP antibody	#60342, proteinintech	1:50	Alexa Fluor-488 conjugated goat anti-mouse IgG (#ab150113, Abcam)	1:500

Supplementary Figure S1. The original data of western blot analysis of Figs. 1m and 1n

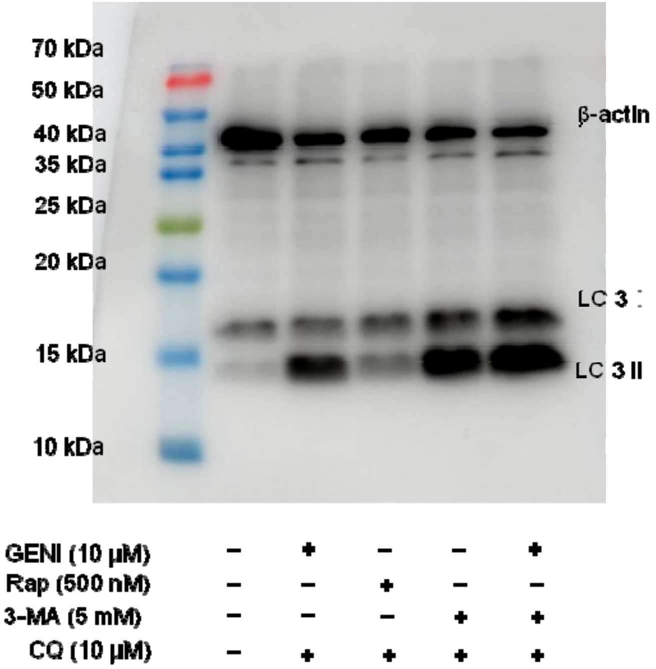

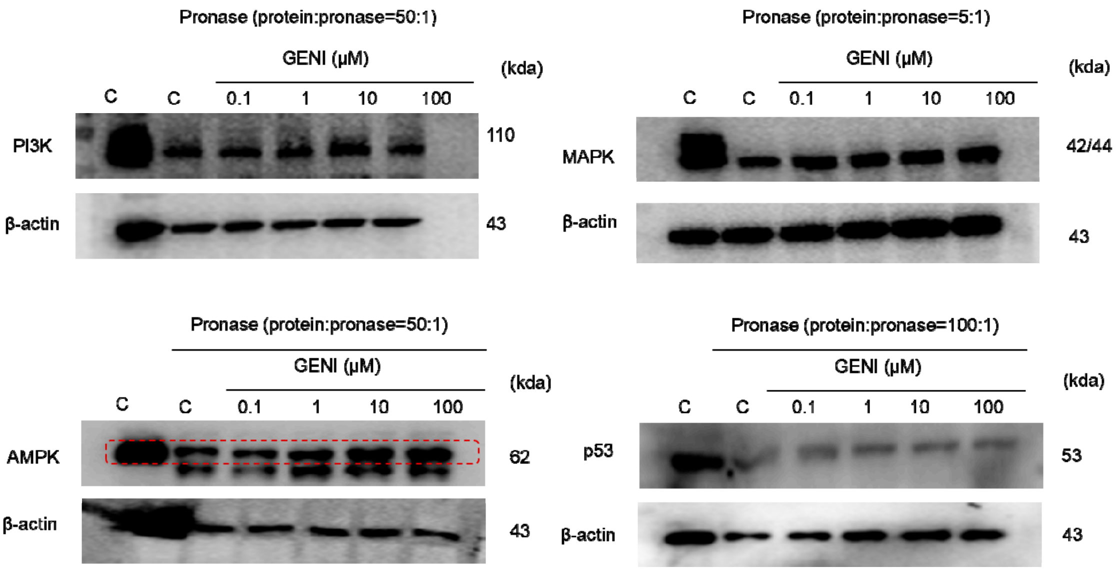

Supplementary Figure S2. The original data of western blot analysis of Figs. 4a-4b

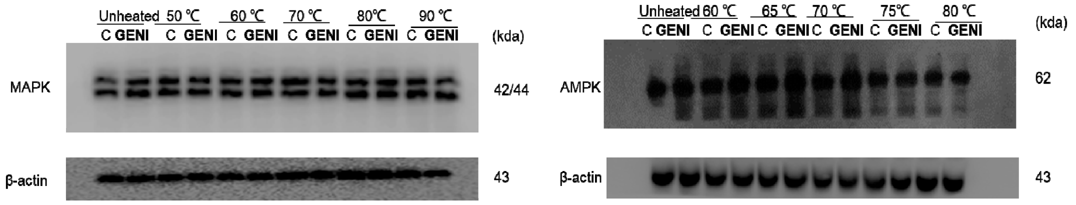

Supplementary Figure S3. The original data of western blot analysis of Figs. 4c-4e

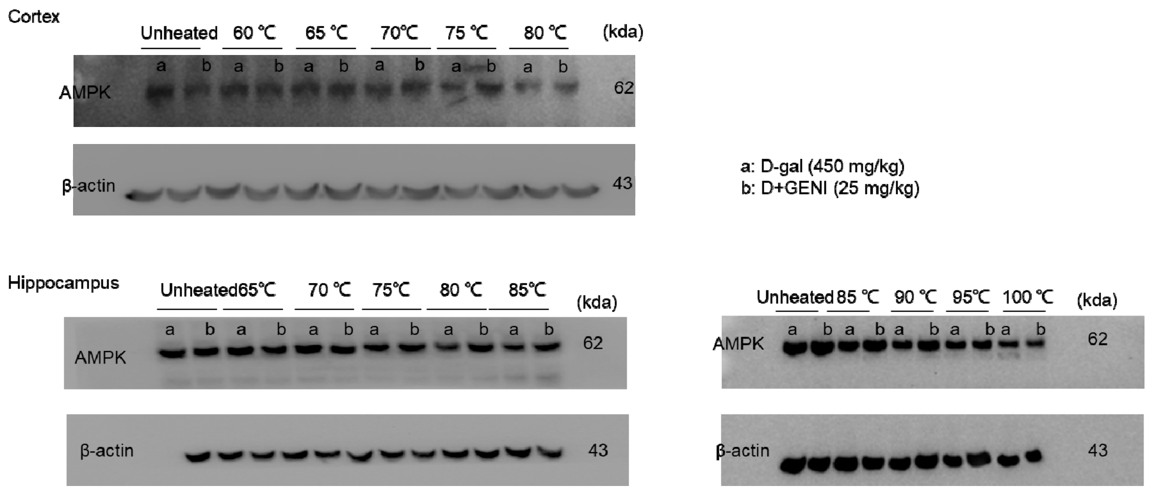

Supplementary Figure S4. The original data of western blot analysis of Figs.4f-4h

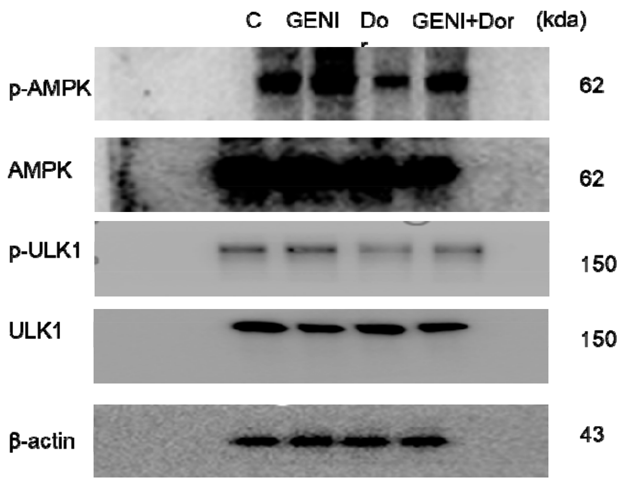

Supplementary Figure S5. The original data of western blot analysis of Figure 4i and 4k

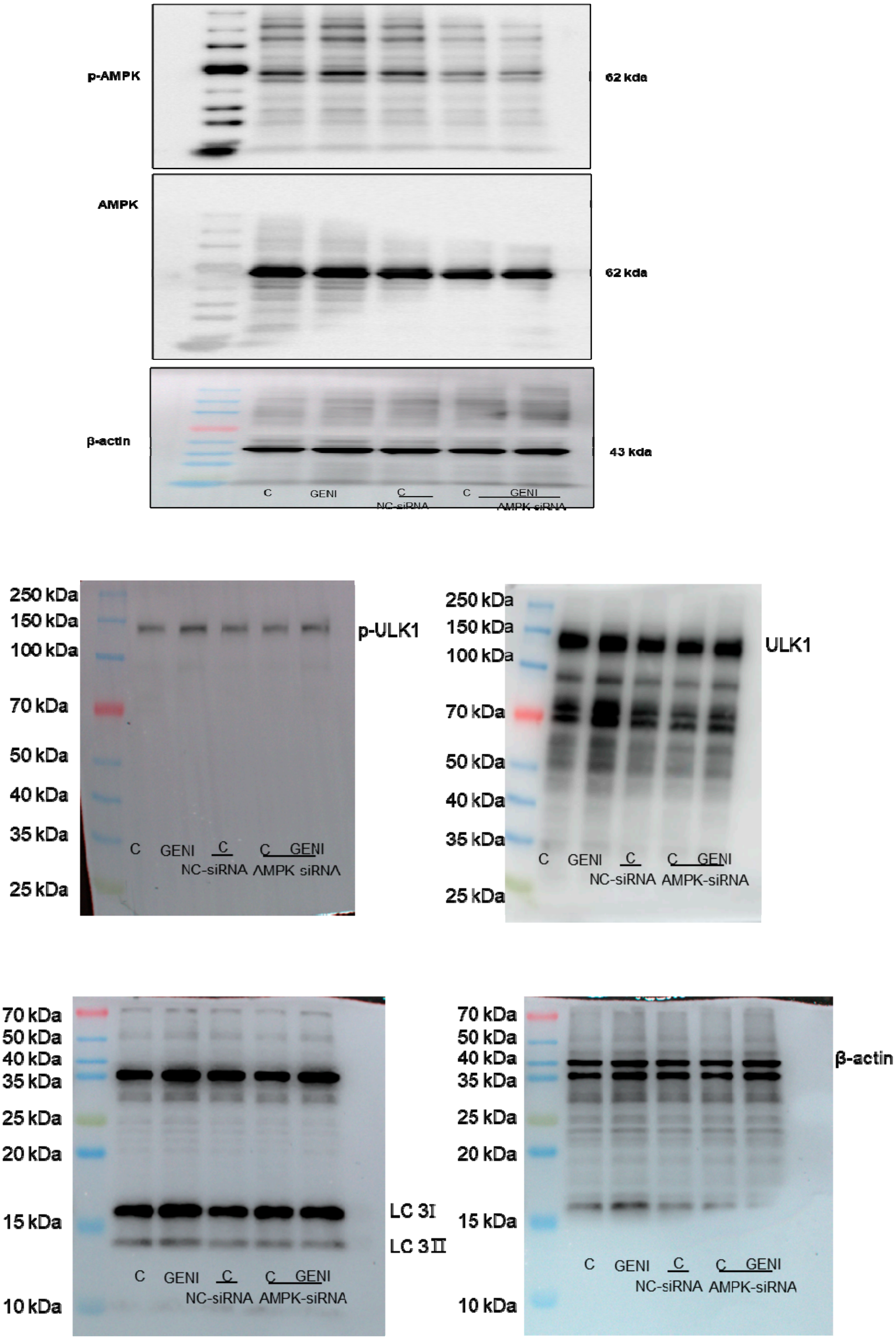

Supplementary Figure S6. The original data of western blot analysis of Figure 4l-4o

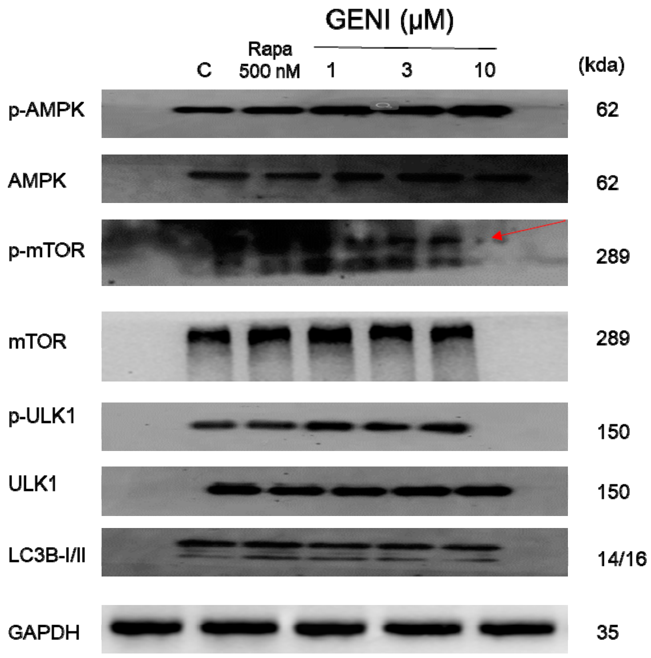

Supplementary Figure S7. The original data of western blot analysis of Extended data Figs. 3a and 3b.

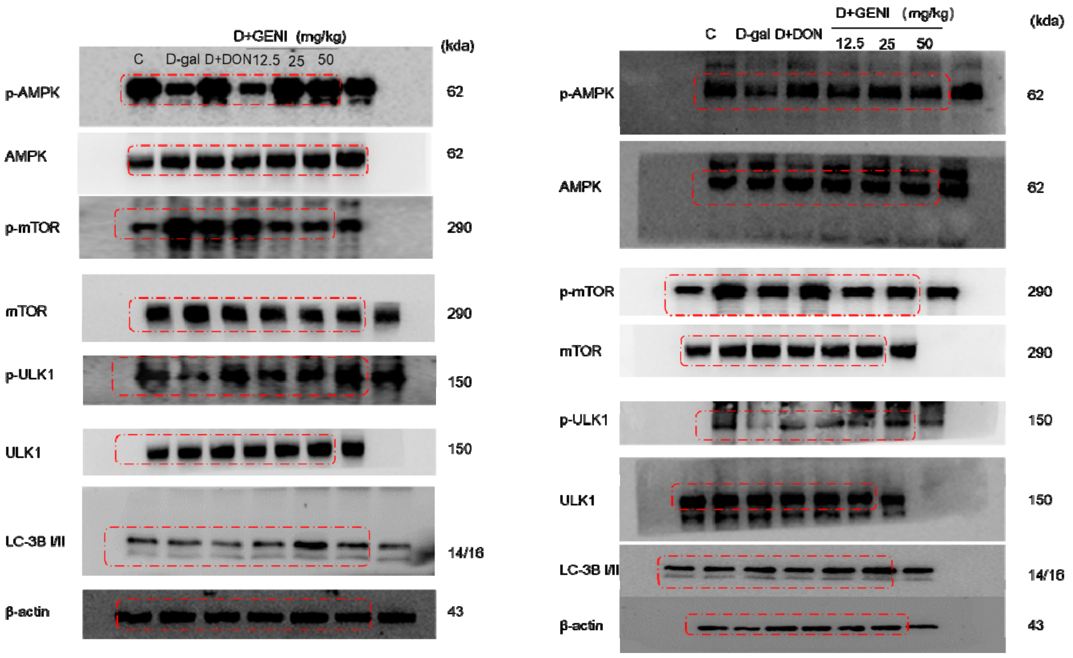

Supplementary Figure S8. The original data of western blot analysis of Extended data Figs. 3c and 3f.

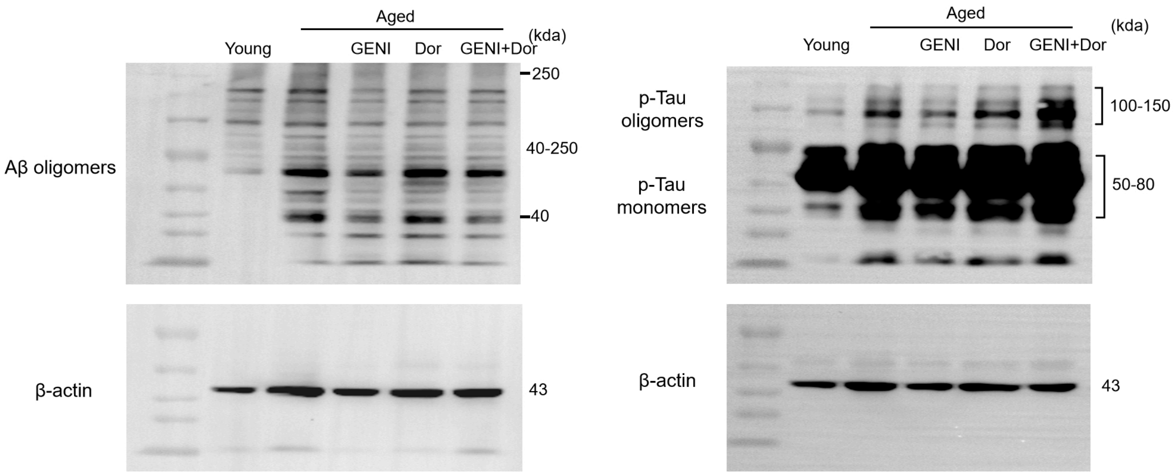

Supplementary Figure S9. The original data of western blot analysis of Extended data Figs. 4e and 4f.

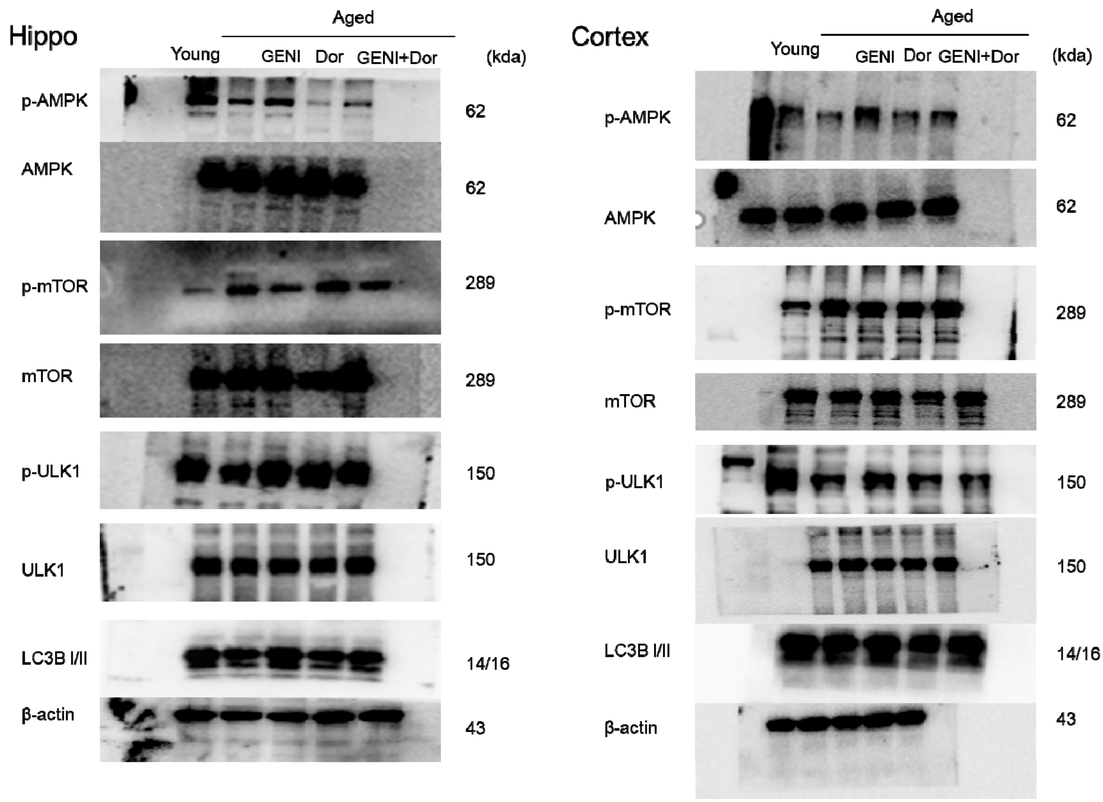

Supplementary Figure S10. The original data of western blot analysis of Extended Data Figs. 5e and 5g.
